# Genetic Variants within SARS-CoV-2 Human Receptor Genes May Contribute to Variable Disease Outcomes in Different Ethnicities

**DOI:** 10.3390/ijms24108711

**Published:** 2023-05-13

**Authors:** Theolan Adimulam, Thilona Arumugam, Anmol Gokul, Veron Ramsuran

**Affiliations:** 1 School of Laboratory Medicine and Medical Sciences, College of Health Sciences, University of KwaZulu-Natal, Durban 4041, South Africa; 215002741@stu.ukzn.ac.za (T.A.); arumagamt@ukzn.ac.za (T.A.); 200003663@stu.ukzn.ac.za (A.G.); 2 Centre for the AIDS Programme of Research in South Africa (CAPRISA), University of KwaZulu-Natal, Durban 4041, South Africa

**Keywords:** COVID-19, SARS-CoV-2, ACE2, TMPRSS2, NRP1, CD147, SNP

## Abstract

The novel severe acute respiratory syndrome coronavirus 2 (SARS-CoV-2) has evolved into a global pandemic, with an alarming infectivity and mortality rate. Studies have examined genetic effects on SARS-CoV-2 disease susceptibility and severity within Eurasian populations. These studies identified contrasting effects on the severity of disease between African populations. Genetic factors can explain some of the diversity observed within SARS-CoV-2 disease susceptibility and severity. Single nucleotide polymorphisms (SNPs) within the SARS-CoV-2 receptor genes have demonstrated detrimental and protective effects across ethnic groups. For example, the TT genotype of rs2285666 (Angiotensin-converting enzyme 2 (ACE2)) is associated with the severity of SARS-CoV-2 disease, which is found at higher frequency within Asian individuals compared to African and European individuals. In this study, we examined four SARS-CoV-2 receptors, ACE2, Transmembrane serine protease 2 (TMPRSS2), Neuropilin-1 (NRP1), and Basigin (CD147). A total of 42 SNPs located within the four receptors were reviewed: *ACE2* (12), *TMPRSS2* (10), *BSG* (*CD147*) (5), and *NRP1* (15). These SNPs may be determining factors for the decreased disease severity observed within African individuals. Furthermore, we highlight the absence of genetic studies within the African population and emphasize the importance of further research. This review provides a comprehensive summary of specific variants within the SARS-CoV-2 receptor genes, which can offer a better understanding of the pathology of the SARS-CoV-2 pandemic and identify novel potential therapeutic targets.

## 1. Introduction

The identification of therapeutic interventions against the COVID-19 pandemic is still ongoing. Despite the lack of definitive treatment, several vaccines have been developed and administered globally [1]. These have varying efficacies, with the highest at approximately 95.0% and the lowest at 66.0% [1]. Despite the relatively high efficacy, SARS-CoV-2 vaccines present with a range of adverse side effects, one of which includes vaccine-induced immune thrombotic thrombocytopenia (VITT), which is fatal [2]. In addition, there are significant differences in disease severity observed across individuals with the same SARS-CoV-2 variants [3]. Consequently, it is crucial to gain more insight into SARS-CoV-2 and its interaction with host cell surface molecules, as this approach may generate novel, unique, and effective strategies for treatment.

The role of polymorphisms within receptors and co-receptors in infectious disease has been previously demonstrated. Human immunodeficiency virus (HIV) infection represents a good model for examining receptor-related mutations. Samson et al. (1996) showed that a mutant allele within the chemokine receptor (*CCR5*) gene conferred significant resistance to HIV infection within Caucasian individuals [4]. The mutant allele (allele frequency of 0.092 in Caucasian populations), now referred to as the CCR5-∆32 HIV-resistance allele, leads to the removal of 32 nucleotides from the *CCR5* gene, resulting in a premature stop codon. This forms a truncated CCR5 protein, which remains in the cytoplasm and does not migrate to the cell surface, preventing the binding of HIV to CCR5 [5]. Interestingly, the CCR5-∆32 mutation is found at a substantially lower frequency within African and Asian populations [6]. Remarkably, bone marrow stem cells with the CCR5-∆32 mutation were successful in curing Timothy Brown of HIV for more than ten years [7,8]. However, it is difficult to reproduce this method. Gupta et al. (2020) recently reported that a London patient who received allogeneic stem cell transplantation with CCR5 absent cells has been in HIV remission for 30 days [9]. The group reported an undetectable viral load in patient plasma. This evidence highlights the importance of determining the effect of genetic variation in viral receptors and their effect on disease susceptibility.

The effect of host receptor polymorphisms can also be observed within the Hepatitis C virus (HCV). The rs5925 SNP of the low-density lipoprotein receptor (*LDLR*) gene was associated with susceptibility in Han Chinese individuals [10]. However, the same relationship was not observed for Japanese individuals [11]. The rs5925 SNP is characterized as a synonymous variant, located within the thirteenth exon of the *LDLR* gene. A second SNP, rs11669576 (1171G>A, Ala391Thr), is a missense variant within exon eight of the *LDLR* gene. Rs11669576 is significantly different in HCV-infected Egyptian individuals compared to healthy controls [12]. In addition, rs11669576 is more frequent in non-responders to an interferon-based treatment as compared to responders (*p* < 0.001) [12]. Other evidence of significant SNPs that influence susceptibility to the disease includes Hepatitis B virus (HBV) infection. Rs2296651 is characterized as a missense variant, resulting in the substitution of serine for phenylalanine at position 267 [13]. Chuaypen et al. (2019) showed that the rs2296651 polymorphism, in the host receptor (sodium taurocholate co-transporting polypeptide (NTCP)) for HBV, influences infection [14]. This study showed that the GA and AA genotypes were associated with decreased risk of HBV infection in Thai individuals [14].

Host receptors and co-receptors are extremely important for viral entry. Disruption of the protein function by SNPs plays a crucial role in disease susceptibility and severity, as demonstrated by HIV, HCV, and HBV. SARS-CoV-2 is similar to these viruses stated above, requiring host receptors and co-receptors for viral replication.

**Figure 1:**
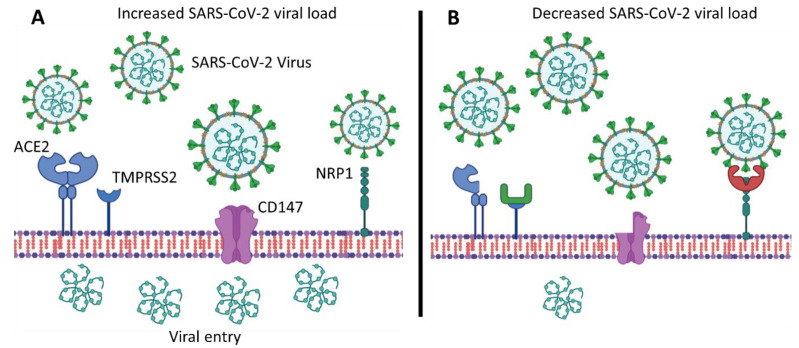
Polymorphisms within *ACE2* (rs2285666), *TMPRSS2* (rs12329760), *NRP1* (rs10080), and *CD147* (rs8259) contribute to variability in SARS-CoV-2 viral load. (**A**) Specific alleles facilitate viral entry and, thus, increased viral load, (**B**) while others disrupt protein function and reduce viral entry into the cell, resulting in lower viral load.

The four main host receptors demonstrated to play a significant role in SARS-CoV-2 infection are ACE2, TMPRSS2, NRP1, and CD147 (Figure 1). SARS-CoV-2 requires the ACE2 receptor to be present on a cell for viral entry, in conjunction with the protein-priming activity of TMPRSS2 [15]. The serine protease TMPRSS2 is key to priming the spike protein of SARS-CoV-2 for interaction with ACE2, as detailed previously [15]. However, subsequent studies have identified additional host cell receptors, facilitating the entry of SARS-CoV-2. A study by Mayi et al. (2021) showed that in vitro viral entry is also dependent on NRP1, a host co-receptor of SARS-CoV-2 [16]. Ke Wang et al. (2020) further showed that CD147 (BSG) is an additional co-receptor for the SARS-CoV-2 virus in hCD147 mice, CD4+ and CD8+ T cells, and human bronchial epithelial cells [17]. In addition, heparan sulphate (HS) glycans facilitate the recruitment of SARS-CoV-2 to the cell surface, increasing localized concentration for effective ACE2 engagement [18]. The variability observed within these genes has the potential to alter COVID-19 disease severity. Diversity in COVID-19 disease severity has been observed across individuals, ethnicities, and even continents [19,20].

The African continent has received significant attention due to the lower SARS-CoV-2 numbers of infections and death reported compared to other continents. At the start of the pandemic, it was predicted that low- and middle-income countries, such as those within the African continent, would be severely affected by the COVID-19 pandemic, mainly due to the poor healthcare infrastructure and the burden of other infectious diseases, such as TB and HIV [21]. Despite the prediction, Africa proved quite the opposite, reporting lower active cases and COVID-19 deaths [22]. In June 2020, the European Centre for Disease Prevention and Control (ECDC), for Africa, reported the lowest number of SARS-CoV-2 infections and deaths (1,037,135 cases and 22,916 deaths) compared to America (10,615,855 cases and 389,793 deaths), Europe (3,061,264 cases and 207,215 deaths), and Asia (4,886,417 cases and 106,711 deaths) [23,24]. Recent studies have described this as the “African paradox” and further identified key features to explain this observation [25,26]. These features include the low median age of the African population, lower testing, the decreased prevalence of co-morbidities, such as obesity and diabetes, the higher rate of helminth infection and malaria incidence [25], demographic structure, lack of long-term care facilities, and public health mitigation strategies [26]. However, these studies neglected to describe the effect of genetic variability within the African population, specifically within the SARS-CoV-2 receptor genes.

In this review, we examine a measure of excess deaths across the world as one indicator of disease severity for the COVID-19 pandemic to limit the bias attached to underreporting across certain countries. From this perspective, we describe previously reported single nucleotide polymorphisms (SNPs) within the SARS-CoV-2 receptor genes (*ACE2*, *TMPRSS2*, *NRP1*, and *CD147* (*BSG*)) to address this African paradox. Therefore, this review provides an in-depth comparison of SNPs within the SARS-CoV-2 receptor and co-receptor genes (*ACE2*, *TMPRSS2*, *CD147*, and *NRP1*) that have a variation in the minor allele frequency higher than 10% between African and European populations.

### 1.1. Inclusion and Exclusion Criteria for Selected SNPs

**Figure 2:**
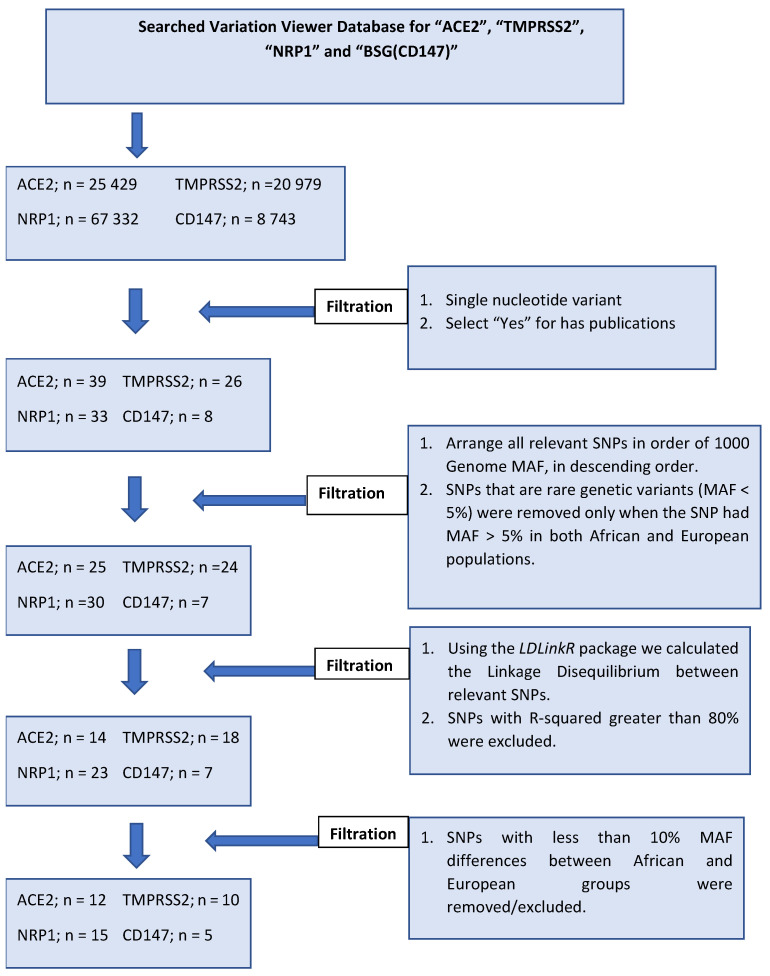
Inclusion and exclusion criteria used to select SNPs discussed in this review for SARS-CoV-2 receptors and co-receptors *ACE2*, *TMPRSS2*, *NRP1*, and *BSG*(*CD147*) genes.

Polymorphisms located within the SARS-CoV-2 receptors and co-receptors were identified from the National Centre for Biotechnology Information (NCBI) (https://www.ncbi.nlm.nih.gov/ (accessed on 20 February 2023)). The SNP database was searched using the search terms “ACE2”, “TMPRSS2”, “NRP1”, and “BSG” under the “genes” category. This search identified multiple SNPs for each gene (*ACE2*, *n* = 25,429; *TMPRSS2*, *n* = 20,979; *NRP1*, *n* = 67,332; and *CD147*, *n* = 8743; Figure 1). These SNPs were further filtered for articles and publications listed on LitVar Annotated, PubMed, and PubMed Linked, decreasing the number of relevant SNPs for each gene (*ACE2*, *n* = 39; *TMPRSS2*, *n* = 26; *NRP1*, *n* = 33; and *CD147*, *n* = 8; Figure 1). SNPs were then ranked in order of minor allele frequencies (MAFs), and SNPs characterized as being rare genetic variants (MAF < 5%) were removed only when the SNP had MAFs less than 5% in both African and European populations (*ACE2*, *n* = 25; *TMPRSS2*, *n* = 24; *NRP1*, *n* = 30; and *CD147*, *n* = 7; Figure 1). Thereafter, the linkage disequilibrium correlation coefficient for each SNP was calculated using the LDlinkR package. SNPs with a correlation coefficient of greater than or equal to 0.8 were removed. Finally, SNPs with greater than 10% MAF difference between Africans and Europeans were selected. Based on these criteria, the following SNPs were discussed for each gene (*ACE2*, *n* = 12; *TMPRSS2*, *n* = 10; *NRP1*, *n* = 15; and *CD147*, *n* = 5; Figure 2 and Table 1).

### 1.2. Excess Deaths Reported between January 2020 and December 2021 of the COVID-19 Pandemic Support the Theory of the “African Paradox”

The African continent possesses the largest genetic diversity compared to the rest of the world [55]. Yu et al. (2002) showed that the average nucleotide diversity is 0.061% ± 0.010% among Asians and 0.064% ± 0.011% within Europeans, with Africans having the largest diversity, 0.115% ± 0.016% [56]. Within African populations, there are higher levels of genetic diversity, large population structure, and lower LD within loci when compared to other populations out of Africa [55]. This genetic diversity within Africa is supported by the magnitude of cultural and linguistic diversity, with over 2000 ethno-linguistic groups throughout Africa [57]. A recent analysis of African (*n* = 426) genomes uncovered an excess of 3 million novel variants and 63 unreported loci within genes responsible for viral immunity, DNA repair, and metabolic function [58]. It has been postulated that variable COVID-19 disease severity can be explained by the genetic diversity in African populations [26]. Recent studies determining the excess mortality associated with the COVID-19 pandemic show marginal differences in excess death when comparing Europe, Asia, and Africa [59,60,61]. Wang et al., 2022 reported on the number of excess deaths due to the COVID-19 pandemic, between 2020 and 2021 [61]. The group collected data for 74 countries and 266 subnational locations and used six models to estimate expected mortality. The study estimated that 18.2 million people died globally compared to the reported 5.94 million [61]. Interestingly, the largest excess deaths due to the COVID-19 pandemic were observed in Asia, North Africa, and Europe [61]. However, Msemburi et al. (2023) estimated 14.83 million excess deaths for the same period. The group reported that America (22%), South East Asia (22%), and Europe (17%) have higher *p*-scores compared to Africa (8%) [60]. With respect to the top 25 countries with the highest total estimated excess deaths (between 2020 and 2021), European and Asian regions contribute 36 and 24 percent, respectively, compared to American and African regions, which both contribute 20 percent [60].

This wealth of evidence suggests that the COVID-19 pandemic was less severe in Africa compared to Europe and Asia. Wachira et al. (2022) and Wamai et al. (2021) recently reviewed the reasons for the lower severity of COVID-19 in Africa [62,63]. These studies explored the low COVID-19 pandemic burden in Africa and suggested that it may be explained by the demographic pyramid, the existence of pre-existing conditions, trained immunity, socio-economic factors, and genetics. To explore the genetic arm, one has to consider the “out-of-Africa” hypothesis. This hypothesis provides an evolutionary theory to suggest that modern-day populations from outside Africa are all primary descendants of a population that left Africa approximately 100,000 years ago [64].

### 1.3. SARS-CoV-2 Receptors, Physiological Significance, and Significant Regulatory SNPs

#### 1.3.1. ACE2

ACE2 is a homologue of ACE and counteracts the negative effect of the renin–angiotensin system (RAS) in a variety of diseases [65,66,67]. ACE2 is physiologically important in vascular regulation. ACE2 functions by reducing the generation of angiotensin (Ang) II by catalyzing the conversion of Ang I to Ang 1–9 and enabling the hydrolysis of Ang II to Ang I-VII. Ang II is the key effector peptide of RAS that causes vasoconstriction. Thus, it is highly expressed in a range of human organs and tissues, such as the heart, kidney, bladder, and intestine. Although the lungs are the primary target of SARS-CoV-2 [68,69], the high expression of *ACE2* in other tissues may be one of the factors contributing to multiple organ injury in critical SARS-CoV-2 infections [70,71].

*ACE2* expression is regulated in different cell types and tissues, via epigenetic mechanisms and polymorphisms, as reported previously [72]. To fully characterize the influence of polymorphisms in the *ACE2* gene, it is worth noting evidence from metabolic diseases, such as hypertension, cardiovascular disease, and diabetes. All of these are associated with COVID-19 comorbidities and linked to exacerbated severity of the disease. Increased *ACE2* expression is indicative of protection against increased blood pressure, while the opposite is also true, where decreased *ACE2* results in hypertension. Patel et al. (2014) found increased ACE2 plasma activity in patients with cardiovascular disease (CVD), as compared to healthy individuals [73].

The ACE2 receptor is used by both SARS-CoV-2 and its predecessor SARS-CoV to enter and infect human cells. Several studies have reviewed the function of the ACE2 receptor and its mechanism of action as a primary receptor for SARS-CoV-2 infection [74,75,76]. Briefly, to infect the human cell, the spike (S) protein found on the envelope of the SARS-CoV-2 virus is cleaved into the S1 and S2 subunits [76]. The S2 subunit has no interaction with the ACE2 receptor; however, S1 contains the receptor binding domain (RBD), which binds directly to the peptidase domain (PD) on ACE2 to infect the human cell [77,78,79]. This interaction is dependent on the proteolytic cleavage of S1 at the C-terminus of the protein, which is achieved by proteases, such as cathepsins and TMPRSS2 [15].

It is, therefore, no surprise that sequence analysis of SARS-CoV-2 and SARS-CoV showed that SARS-CoV-2 clusters with SARS-CoV-related viruses [15]. Zhou (2020) and colleagues showed that the presence of the ACE2 protein is required for viral entry, and cells that do not have this protein remain uninfected [80]. Furthermore, cells that possess other coronavirus receptors, such as aminopeptidase N (APN) and dipeptidyl peptidase 4 (DPP4), are not infected with SARS-CoV-2. Hoffman et al. (2020) also showed that the receptor binding domain (RBD) of the spike protein was conserved between SARS-S and SARS-2-S, which further validates the findings of Zhou and colleagues [15,81].

Due to the role of ACE2 in SARS-CoV-2 infection, studies have investigated whether *ACE2* expression and function is affected by polymorphisms. Li et al. (2005) showed that changes in the ACE2 protein are key for the binding of SARS-CoV [82]. Thus, genetic variability within the *ACE2* gene may contribute to the ability of SARS-CoV-2 binding to ACE2, providing an explanation for the variability in COVID-19 susceptibility and disease severity across different ethnic groups.

Before the COVID-19 pandemic, studies identified associations between malaria and increased blood pressure. Further investigation into the association revealed that specific polymorphisms found in malaria-endemic regions provided a protective effect towards malaria severity. The first study to investigate the association of *ACE* insertion/deletion (I/D) within intron 16 with malaria found that the *ACE* I/D polymorphism, responsible for increased Ang II production, was significantly associated with mild malaria in India (*p* < 0.0001). The study showed that the D-allele, in both the homozygous (DD) and heterozygous (DI) form, was significantly associated with mild malaria, suggesting that the D-allele has a protective effect against susceptibility to severe malaria [83]. Rigat et al. (1990) found that the presence of the D-allele was associated with increased ACE enzymatic activity compared to the I-allele [84]. Dhangadamajhi et al. (2010) also investigated the association of *ACE2* C>T polymorphism located in intron 1. The found that individuals with at least one T-allele (i.e., TT or CT) were more likely to be protected against malaria in women. The gender-specific effect showed that the T-allele was associated with lower expression of *ACE2* [83].

Protection against severe malaria has been associated with polymorphisms in genes that regulate Ang II. Higher levels of Ang II are a major characteristic of hypertension, as observed in African and South Asian populations, which present with elevated blood pressure [85,86]. ACE2 cleaved Ang I, forming the Ang II vasopressor, consequently increasing blood pressure [87]. Therefore, even though higher levels of Ang II are associated with hypertension and mild malaria, this suggests that lower levels of ACE2 are present in African and South Asian populations [88]. Interestingly, rs1514282 (T>C) (Table 1) has been associated (*p* < 0.006) with mean arterial pressure during high-sodium interventions within East Asian individuals [29]. Recent in silico analysis suggested that the wild-type allele of rs1514282 results in binding of the elav-type RNA binding protein (ETR-3), which may induce exon inclusion within *ACE2* [89].

This can be further extrapolated to account for the lower severity of the COVID-19 pandemic observed in Africa. Therefore, the hypothesis that links malaria–hypertension–COVID-19 is rational, further warranting the need to identify *ACE2* polymorphisms, such as rs2106809 (A>G), rs2285666 (C>T), and others, which control the expression of *ACE2* [90]. Since the COVID-19 pandemic, various studies have outlined the relationship between malaria–ACE2 and COVID-19 severity and susceptibility [90,91,92,93].

The most characterized *ACE2* SNP, the splice variant rs2285666 (C>T) in intron 3, has been previously associated with COVID-19 comorbidities [94]. The A-allele is associated with increased serum levels of ACE2 in individuals with diabetes and cerebral stroke [95]. Despite the wealth of evidence of increased plasma and serum levels of ACE2, it is unknown whether these levels correlate with membrane expression of the ACE2 receptor. If plasma and serum levels of ACE2 share a positive correlation, this could be used as a method to predict the susceptibility and severity of SARS-CoV-2. However, this needs to be further evaluated. Studies investigating the roles of rs2285666 and rs2106809 in SARS-CoV-2 infection and disease severity have identified contradictory results [96,97,98].

Molina et al. (2022) showed that rs2285666 was significantly associated with COVID-19 disease severity in Spanish individuals [96]. Furthermore, rs2285666 was associated with an increased risk of hospitalization (OR = 6.65, *p* = 0.048) in females from a Spanish cohort [96]. However, analysis of a Turkish cohort found no association between this SNP and the severity of COVID-19 [98]. Despite this, recent data showed that the T-allele of rs2285666 had an increased frequency in mild and severe SARS-CoV-2-infected individuals, further highlighting its effect on COVID-19 disease severity [99]. In addition, Alimoradi et al. (2022) concluded that the GG genotype of rs2285666 was not associated with COVID-19 severity but with infection instead [97]. This conclusion is highly speculative, owing to the difficulty in defining a SARS-CoV-2 true negative. Despite qPCR being the gold standard for SARS-CoV-2 testing, there is no way of determining if an individual has had significant exposure for infection to occur.

In silico analysis identified rs2106809 as a causal SNP for the creation of an intronic-exonic splicing enhancer site in individuals with a CC or CT genotype having higher circulating levels of ACE2 [98]. These data support the association of rs2106809 with disease severity in Spanish individuals and the increased risk of hospitalization (OR = 2.12, *p* = 0.039) [96]. However, Karakaş Çelik et al. (2021) reported no association with COVID-19 disease severity within a Turkish cohort [98]. The contrasting results of these studies can be explained by the smaller sample size and differences in cohort demographics.

Rs1978124 (T>C) is an intron variant with a higher frequency in African compared to European individuals (0.90 and 0.53, respectively). The A-allele has been previously associated with lower systolic function in Caucasian men with Type-2 diabetes (T2D) and cardiovascular disease [100]. This was further validated to be significantly associated with cardiovascular disease in T2D Uygurs (Asian) [27]. In addition, the T-allele of rs1978124 (*p* = 0.009) and rs233575 (*p* = 0.018) was associated with dyslipidemia in an Asian population [101]. In addition to rs233575 (G>A) being previously associated with dyslipidemia, it has recently been associated with hypertension in French-Canadian obese males, suggesting a risk of severe COVID-19 [102]. Rs1978124 has been associated with contrasting effects on COVID-19 disease severity. Molina et al. (2022) showed that rs1978124 was significantly associated with disease severity in Spanish individuals [96], while Faridzadeh et al. (2022) reported that the TT and CT genotype had a significantly positive role in SARS-CoV-2 susceptibility and a protective effect on disease severity in Iranian females [103].

Rs35697037 has been characterized as being significantly associated with dizziness [34]. Wang et al. (2014) showed that the AA and AG genotypes were associated with increased incidence of dizziness (*p* = 0.0122) within Asian individuals [34]. In conjunction with medical conditions, such as blood pressure and cardiovascular diseases, dizziness has been described for COVID-19 neurotropism [104,105]. Jafari et al. (2021) found the occurrence rate of dizziness was at 12.20% in SARS-CoV-2-infected individuals [106]. The evidence of dizziness in SARS-CoV-2-infected individuals has only been reported in a few case reports [107,108,109]. It is possible that rs35697037 (G>A) may be prevalent in COVID-19-positive individuals who experienced dizziness as a symptom; however, this assumption requires further investigation.

A recent study found that COVID-19 is associated with an increased risk of acquiring diabetes in American individuals (OR 2.56) [110]. The incidence of diabetes 120 days after infection was higher in Black (OR 1.61) individuals compared to Europeans (OR 1.09) [110]. Liu et al. (2018) identified an association of rs879922 (C>G) with T2D (*p* < 0.05) among East Asians, suggesting the SNP may be a common marker for susceptibility to T2D [27]. Rs879922 is found at a lower frequency in African (45%) individuals compared to Europeans (65%). Jalaleddine et al. (2022) showed that rs879922 was associated with COVID-19 in obese compared to lean individuals (*p* = 0.06), which may increase COVID-19 disease severity [111]. In addition, rs2048683 (T>G) was associated with T2D in East Asians [111] and blood pressure in European individuals (2020). Elbadri et al. (2022) reported that the GG genotype (OR 5.852) is a potential marker for the risk of severe SARS-CoV-2 infection within Egyptian individuals [112]. Furthermore, this study reported a significant association between the TT and GT genotypes (*p* < 0.001) of rs2048683 with T2D [112]. Together, these SNPs may be used as potential markers for T2D and risk of severe SARS-CoV-2.

The CC genotype of rs714205 (C>G) has been shown to be associated with diabetic retinopathy (*p* < 0.05) in Asian individuals [35], while the GG genotype was associated with an increased risk of hypertension [113].

In a recent study, population-specific SNPs were identified, which found significant differences in African individuals [114]. The same group identified 13 *ACE2* variants that facilitated better interaction between ACE2 and viral S1. These include rs142984500 (H378R) and rs7363825 (S19P), which were identified as being specific to European (*p* < 0.0449; frequency: 0.00014) and African populations (*p* < 0.0000; frequency: 0.0033) [114]. In addition, the study identified 18 SNPs that were characterized as interaction-inhibitory variants. Interestingly, the African population had the highest difference in MAF frequency of these SNPs compared to other ethnic groups. For instance, rs766996587 (M82I) is an African-specific variant (*p* < 0.0345; frequency: 0.00016) [114].

In addition to these SNPs, rs2097723 (T>C) is found at lower frequency in Africans (7%) compared to East Asian (42%), European (28%), and Asian (22%) individuals. Notably, the C-allele is proposed to increase *ACE2* expression in the brain tissue, and it may be a driving factor for COVID-19 disease severity within East Asians [115].

Collectively, these studies highlight the genetic similarity between Asians and Europeans. Phylogenetic analysis by Srivastava et al. (2020) identified a rs4646120 (G>A) and rs2285666 haplotype unique to South Asians and East Eurasians, which may contribute to similarities in COVID-19 susceptibility [33]. In addition, *ACE1* and *ACE2* haplotypes have been identified and attributed to the disproportionate effect of the COVID-19 pandemic on Europeans and Asians [116]. Gemmati et al. (2020) suggested that rs2285666, rs1978124, and rs714205 may be predictive markers for COVID-19 disease severity [116]. However, the use of these SNPs as molecular markers for disease severity requires further investigation due to the differences observed across different ethnic groups.

Within the *ACE2* gene, rs1978124, rs233575, rs1514282, rs16997078, rs2048683, rs4646120, and rs4646140 are found to at higher frequency in African individuals (Table 1), while rs2097723, rs879922, rs2106809, rs35697037, and rs714205 have a higher frequency in European individuals. Interestingly, rs1514282, rs16997078 (A>G), and rs4646140 (C>T) are unique to African individuals compared to Europeans. Studies within an Asian population have shown the influence of the intronic SNPs (rs4646140 and rs35697037) on hypertension. Despite the higher frequency of these polymorphisms in African individuals, research on these polymorphisms within Africa is lacking [31,35]. A recent in silico analysis of *ACE2* revealed that 15 intronic and 2 missense SNPs (rs147311723 and rs149039346) were different in African individuals compared to other populations [117]. Several other studies have highlighted these SNPs as being involved in COVID-19 severity and genetic susceptibility [94,115,118,119].

#### 1.3.2. TMPRSS2

TMPRSS2, a cell surface trypsin-like protease, has been identified as a key mediator of viral infection. On the plasma membrane, TMPRSS2 proteolytically cleaves and activates viral S glycoproteins, enabling the entry of viral particles [120]. As such, TMPRSS2 has been implicated in the spread and pathogenesis of influenza A virus, human corona virus 229E, human coronavirus EMC, Sendia virus, human metapneumovirus, and human parainfluenza [120,121,122,123]. Thus, it is not surprising that TMPRSS2 is an essential co-receptor for the pathogenesis of SARS-CoV-2 [124]. The primary role of TMPRSS2 in SARS-CoV-2 infection has been described as proteolytic cleavage of the S2 subunit, thus triggering the fusion of the viral envelope with the cell membrane [15,125,126].

Apart from its well-described microbial activity, the normal physiological function of TMPRSS2 remains unknown. It is, however, highly expressed in the prostate compared to other human tissue and facilitates prostate cancer cell metastasis [127,128]. Lubieniecka et al. (2004) showed that the GG genotype of rs1232970 and a family history of prostate cancer increased the risk of prostate cancer acquisition [129]. Therefore, it is worth investigating the effect of SNPs on COVID-19 disease susceptibility and severity.

The assessment of SNPs within *TMPRSS2* showed that rs2070788, rs8127290, rs383510, rs11910678, and rs1557372 are found at higher frequency in African individuals, while rs463727, rs35041537, rs7275220, rs456298, and rs75603675 are found at higher frequency in European individuals (Table 1). Wang et al. (2020) investigated single-nucleus-accessible chromatin profiles to determine susceptibility to SARS-CoV-2 infection and compared common variants with infection and respiratory function [130]. The study showed that rs8127290 (G>A) (*p* = 1.4 × 10^−3^) and rs1557372 (C>T) (*p* = 2.9 × 10^−3^) were associated with asthma and chronic obstructive pulmonary disorder, respectively, which may influence COVID-19 susceptibility and severity [130].

Based on eQTL data, Asselta et al. (2020) showed that rs463727 (T>A) significantly changed *TMPRSS2* expression between European and East Asian individuals (*p* < 2.2 × 10^−16^) [131]. Rs463727 and rs35041537 (C>T) are found at a lower frequency within an Asian cohort, which may explain the low rate of SARS-CoV-2 infectivity within an Indian cohort [37].

Rs11910678 (T>C) has a higher frequency in African individuals compared to European individuals (Table 1). A study within an Asian cohort found that rs11910678 influenced *TMPRSS2* expression [42]. This same effect has not been explored within an African setting.

In males, rs8134378 (A>T), rs383510 (T>C), and rs2070788 (G>A) have been shown to increase the expression of *TMPRSS2*, favoring the fusion of H1N1 and H7N9 viral membranes [40]. In summary, *TMPRSS2* expression is influenced by the G-allele of rs2070788 and the T-allele of rs383510, which are collectively associated with elevated *TMPRSS2* expression in lung tissue [132]. Rs2070788 and rs383510 have been further identified as causal SNPs of COVID-19 disease severity [133]. Panday et al. (2022) showed that the G-allele of rs2070788 was significantly correlated with the case fatality rate of the Indian population (*n* = 393; *p* = 0.029) [134]. However, Schönfelder et al. (2021) did not correlate rs2070788 with increased risk or disease severity of SARS-CoV-2 infection within a German cohort (239 positives and 253 negatives) [135]. Interestingly, the G-allele of rs2070788 is the same in both Asians and Europeans; thus, the differences in results can be attributed to different study designs. Pandey et al. (2022) considered next-generation sequencing data against the COVID-19 case fatality rate across India [134], while Schönfelder et al. (2021) compared 239 SARS-CoV-2-positive individuals and 253 SARS-CoV-2-negative individuals who had typical symptoms for COVID-19 but did not test positive via RT-PCR [135].

Despite the relatively higher frequencies of rs2070788 and rs383510 in Africa, the impact of these SNPs has not been explored. In addition, rs2070788, rs9974589, and rs7364083 have been predicted to be associated with higher *TMPRSS2* expression in European individuals [131]. Investigating these SNPs in an African population would discern their association with severe COVID-19.

Among the various SNPs that have been associated with COVID-19, rs12329760 has been identified to exhibit both a deleterious and protective effect, specifically within young males and older females [136]. The SNP is characterized as a missense variant with the substitution of valine with methionine at position 160 (c.478G>A, p.V160M). Wulandari et al. (2021) identified a weak correlation between rs12329760 and viral load within individuals of Indonesian descent, indicative of an association with SARS-CoV-2 infectivity and disease severity [137]. In addition, Abdelsattar et al. (2022) showed that the T-allele of rs12329760 is significantly increased in a severe COVID-19 Egyptian group [99], while these studies have identified strong correlations of rs12329760 with increased COVID-19 disease severity. Ravikanth et al. (2021) demonstrated that rs12329760 was associated with decreased disease severity [138]. In addition, a 2.5-fold increase in mRNA and protein expression was observed in individuals with the variant, and a 2.4-fold decrease in spike protein cleavage was observed in individuals with the variant [138]. Recent data from the UK further highlight the protective effect of the T-allele [139]. A group showed significant data for individuals of European and East Asian ancestry (*p* = 0.01; OR = 0.87 and *p* = 0.03; OR = 0.64, respectively); however, no significant data were obtained for African and South Asian ancestries due to low sample numbers [139]. Despite the pronounced protective effect of rs12329760 within European individuals, Schönfelder et al. (2021) did not report a correlation between rs12329760 and COVID-19 [135].

While the role of rs75603675 (C>A) within infectious diseases is largely unknown, Torre-Fuentes et al. (2021) found that the SNP was more frequent among SARS-CoV-2-infected Spanish individuals; however, the data were not significant, owing to the small sample size of 138 individuals [140]. On the contrary, Iranian data suggest that the AA genotype of rs75603675 decreased the risk of severe COVID-19 (*p* = 0.027) [141]. Furthermore, Villapalos-Garcia et al. (2022) identified rs75603675 as a possible predictor for severe disease from 817 participants of Eurasian descent (OR = 2.140) [142]. The lower frequency of this SNP in African individuals may be a concerning predictor for disease severity.

In a recent study, Posadas-Sanchez et al. (2022) investigated SNPs within TMPRSS2 within a Mexican cohort [39]. Their study of rs2298659 (G>A), rs456298 (T>A), rs462574 (A>G), and rs12329760 (C>T) resulted in two high-risk haplotypes (ATGC and GAAC) and, interestingly, a further two protective haplotypes (GAGC and GAGT). The results of this study point to the probability of the same SNPs being high-risk alleles and low-risk alleles. Based on this observation, it is worth noting that, irrespective of an SNP being a marker for a high risk of COVID-19 in one population, the same SNP may be highly protective in another population.

Collectively, these studies highlight the protective and detrimental effects of the *TMPRSS2* polymorphisms within various ethnic groups. However, this has not been proven for individuals of African ancestry. We suggest that the same protective effect may be present in African populations, owing to the relatively higher frequency of the rs12329760 T-allele compared to European populations. However, this needs to be further investigated within an African setting.

#### 1.3.3. NRP1

NRP1 is a cell surface receptor involved in angiogenesis, vascular permeability, and the development of the nervous system. NRP1 is a receptor for vascular endothelial growth factor (VEGF) that controls the binding of VEGF to kinase insert domain receptor (KDR), thus regulating VEGF-induced angiogenesis [143,144]. In addition, NRP1 is a mediator of chemorepellents by interacting with Collapsin-1/Semaphorin III/D [145]. The most significant function of the NRP1 receptor is its ability to recognize and bind to C-end Rule (CendR) peptides, which facilitates the entry and transport of peptides through tissues [146].

This characteristic has been shown to enable the recognition and binding of the CendR motif RRAR on the Spike 1 protein of SARS-CoV-2 [147,148]. Ackermann et al. (2020) showed that the expression of *NRP1* was increased in SARS-CoV-2-infected patients [149]; thus, polymorphisms that influence *NRP1* expression may play a regulatory role in the further uptake of viral particles. Interestingly, it has been suggested that NRP1 may interact with S1 in the absence of ACE2; however, this requires further investigation [150].

Rs2228638 (C>T) and rs10080 (G>A) have both been identified as polymorphisms that may impact the clinical outcomes of SARS-CoV-2 infection [151] (Table 1). Fan et al. (2018) demonstrated that rs2228638 is associated with an increased risk of tetralogy of Fallot (TOF) in a Chinese population (*p* = 0.002) [48]. Tetralogy of Fallot is a severe form of cyanotic congenital heart disease, where the T-allele has been associated with increased risk in European and Chinese populations [48]. In addition, rs10080 is also associated with susceptibility of TOF (*p* = 0.001). The association of these SNPs with severe clinical outcomes of COVID-19 is, thus, justified, owing to the association of cardiovascular disease with severe COVID-19 disease.

A major symptom of SARS-CoV-2 infection is headaches [152]. Interestingly, an *NRP1* SNP, rs2506142, is associated with migraine susceptibility and significantly associated with menstrual migraine (*p* = 0.003) [153,154]. Although this SNP does not associate with disease susceptibility, it is worth noting the effect this SNP may have on clinical manifestations and severity of COVID-19 disease.

Within *NRP1*, rs927099, rs1048804, rs10080, rs1571781, rs2070296, rs2506144, rs1888686, and rs1010826 have a higher frequency in African individuals. Studies of these polymorphisms within other ethnic groups are lacking. On the contrary, rs2804495, rs1319013, rs12573218, rs1331326, rs1888685, rs12358370, and rs2228638 have a higher frequency in European individuals.

#### 1.3.4. BSG (CD147)

CD147, more commonly known as Basigin, is a transmembrane glycoprotein within the immunoglobulin superfamily [155]. CD147 has well-characterized associations with tumor development, *Plasmodium* invasion, and bacterial and viral infections [156,157,158,159]. Chen et al. (2005) proved the functional role of CD147 in cellular invasion of SARS-CoV and HIV-1 [160]. The evidence from this previous work highlighted the role of CD147 in SARS-CoV-2 infection. Wang et al. (2020) discovered CD147 as a new receptor for the SARS-CoV-2 virus [17]. Interestingly, the group found that ACE2-deficient T cells (BHK-21 cells) could be infected with the SARS-CoV-2 pseudo virus. The overexpression of CD147 facilitates viral infection but also alters viral tropism.

Despite this evidence, Shilts et al. (2021) were unable to validate the role of CD147 in SARS-CoV-2 infection in HEK293 cells [161]. The group did not detect any biochemical binding or cell-based assay interaction with CD147 and the SARS-CoV-2 spike protein [161]. Shilts et al. (2021) further explored the functional role of CD147 in lung epithelial cells (CaLu-3), where CD147 had no significant effect on COVID-19 infectivity, despite ACE2 receptor knock-out using CRISPR-Cas9. These contrasting results are attributed to the different in vitro models employed. This highlights the cell-specific nature of the gene.

Considering these conflicting reports, Fenizia et al. (2021) sought to further test the hypothesis that CD147 may be involved in SARS-CoV-2 infection through interaction with cyclophilin A (CyPA) [162]. CyPA is a member of the immunophilin family, which promotes viral infection [156,160] and has known interactions with CD147 [160]. Contradictory to the report by Shilts et al. (2021), Fenizia et al. (2021) found that knock down of *CD147* via transient siRNA transfection significantly decreased pulmonary cell viral load but also decreased the availability of the ACE2 protein [161,162]. The results support the initial discovery by Wang et al. (2020); however, they also suggest that CD147 may influence SARS-CoV-2 infection, either directly or indirectly, due to *ACE2* regulation. Fenizia et al. (2021) further showed that *CD147* silencing decreased ACE2 protein levels (82.3 ± 7.0 percent) but did not affect RNA expression [162].

Thus, the role of CD147 in SARS-CoV-2 infection has since been controversial. We suggest that the use of different cellular models, in vivo, as well as molecular knock out techniques, can account for the conflicting data. However, recent data support the role of CD147 in SARS-CoV-2 infection [163].

The most studied SNP within *CD147* is rs8259 (T>A), which is associated with susceptibility of psoriasis [52] (Table 1). Wu et al. (2011) showed that rs8259 was in the seed region of miR-492, resulting in the selective binding of miR-492 [52]. The A-allele was associated with no effect of binding miR-492, while the T-allele facilitated miR-492 binding. The group reported that miR-492 may have the potential to bind to and inhibit the expression of *CD147* within individuals expressing the TT-genotype (*p* = 0.027) [52]. Furthermore, rs8359 is associated with acute coronary syndrome [164]. Congruent with previous reports, Mao et al. (2014) showed that the AA-genotype increased mRNA and protein expression of CD147 in plasma [164]. The group did not explore the effect of miR-492; however, it is worth noting that the miRNA may be implicated in controlling the expression of *CD147*. Other studies have associated rs8592 with the risk of chronic heart failure [165] and multiple myeloma. The TT-genotype was found to confer a decreased risk of chronic heart failure in Chinese individuals (*p* = 0.010) with hypertension and chronic heart disease [165]. This SNP has been extensively explored within the Chinese Han population; studies in African and European populations are undefined.

A recent study by Latini et al. (2020) identified three SNPs of interest with the *CD147* gene, which may be involved in SARS-CoV-2 entry into cells [166]. The SNPs include rs201850688, rs11551906, and rs144824657, which do not reflect differences in the MAF between ethnic groups. Crosnier et al. (2011) identified key SNPs that modulate *NRP1* interaction with *Plasmodium falciparum* [167]. Interestingly, rs104894669 resulted in a 2-fold reduction in parasite up-take, while rs55911144 resulted in a loss of interaction between NRP1 and the parasite. However, these SNPs have no variation in MAF between ethnic groups.

Furthermore, studies have identified causal SNPs within *CD147*, which have been associated with coronary heart disease and acute myeloid leukemia [51,53]. These include rs8637, rs8259, rs4919862, rs4919859, and rs6758, which have been previously identified for possible impact on COVID-19 severity [151]. However, despite the array of knowledge on cancers and other infectious diseases, the role of *CD147* polymorphisms with SARS-CoV-2 infection is largely unknown [151,168].

SNPs in *NRP1* and *CD147* may be potential therapeutic targets. Studies suggest that the use of NRP1 and CD147 inhibitors may be an alternative COVID-19 therapeutic strategy [162,169].

Despite the high frequency of rs2804495 (G>T) in Europeans, Lores-Motta et al. (2016) did not report an association with treatment response to neovascular age-related vascular degeneration [45]. Studies to investigate the role of rs2804495 in COVID-19 have not been considered.

### 1.4. Addressing the African Paradox

The COVID-19 pandemic has demonstrated variable effects in different countries around the world. Several studies have characterized and explained the African paradox through socio-economics effects and low mean age. However, the genetic effects of COVID-19 disease susceptibility and severity are not yet fully understood within an African setting. This review highlights the lack of studies within Africa regarding the COVID-19 pandemic, which will contribute to the understanding of the African paradox. Africa has made a lower contribution to the COVID-19 Host Genetics Initiative as compared to Europe and America [170]. Furthermore, the African genome is widely understudied in comparison to European and Asian populations. Thus, intense studying of the African genome will reveal genetic factors that contribute to protection from infectious diseases. Understanding genetic variation within African populations may help curb the disparity of SARS-CoV-2 infection by providing critical pieces of information that can be used for the development of potential therapeutic markers, also highlighting the importance of personalized treatment strategies.

This review highlights several differences in the association of genetic variants within the SARS-CoV-2 receptor genes and COVID-19 outcomes across ethnic groups. However, a limitation of this approach is the variability in sample sizes in case–controls and research methodologies across studies. This is exemplified by the results obtained by Schönfelder et al. (2021), who reported contrasting results for both rs2070788 and rs12329760 compared to other studies.

### 1.5. SARS-CoV-2 Receptor-Based Therapy and Clinical Significance

Several therapeutic strategies for COVID-19 have been suggested, which primarily target S1-ACE2 interaction. Mostafa-Hedeab, 2020, suggested the use of an ACE2 ligand or antibody to block the receptor binding domain of ACE2 to prevent SARS-CoV-2 infection [171]. Recently, the administration of recombinant soluble human ACE2 (rhACE2) in combination with remdesivir has been shown to block the entry of SARS-CoV-2 into cells, acting as a “decoy” for the ACE2 receptor [172]. Currently, rhACE2 APN01 is in phase two of clinical trials (NCT04335136). In addition to ACE2, NRP1 inhibitors have been suggested as possible therapies [169]. In silico screening of possible NRP1 inhibitors showed that Nafamostat, Y96, Selinexor, Ebastine, and UGS are possible drugs, which may inhibit S1-NRP1 interactions [173]. Yamamoto et al. (2020) showed that Nafamostat inhibits SARS-CoV-2 entry in human lung cells [174]. Furthermore, Selinexor is currently being used in two phase two clinical trials (NCT04349098 and NCT04355676); however, cancer patients receiving Selinexor therapy in clinical trials developed infections during the trial [175]. The need for alternative therapeutic targets and strategies spurs on further research and understanding of the SARS-CoV-2 receptors.

This study further highlights the potential role of variants with similar MAFs for the use of biomarkers for COVID-19. Yagin et al. (2023) recently developed artificial intelligence model (extreme gradient boosting) gene expression profiles, capable of successfully predicting COVID-19 [176]. Similarly, SNPs should be studied for their potential use as predictive biomarkers for COVID-19 severity. This provides clinicians with an advanced tool for the prediction of higher or lower COVID-19-risk individuals, thus targeting vaccination role out and rapid therapeutic interventions at higher-risk individuals.

## 2. Conclusions

This review highlights the effect of host genetics within SARS-CoV-2 receptors that play a role in the pathogenesis of disease. We reviewed 42 SNPs within *ACE2* (12), *TMPRSS2* (10), *CD147* (5), and *NRP1* (15), and we discussed their role in other infectious diseases and non-communicable diseases. Furthermore, we highlighted the importance of genetic polymorphisms within these receptors, which may influence disease susceptibility and severity across different ethnicities. We highlighted the need for in-depth study of African genetic diversity across infectious diseases. When revieing the literature, we found that studies within European and Asian cohorts outweigh studies from an African perspective. Research has relied heavily on 1000s of genomes to determine the frequency of these polymorphisms in African populations, but without further studies, we do not know the relevance of these SNPs within Africans and their impact on disease. We also noted major differences in the frequency of SNPs within African vs. European and Asian individuals, which may explain the disparity of COVID-19 across ethnic groups. This review supports the theory of the “African Paradox”, due to the major differences of SNPs within the SARS-CoV-2 receptor genes and the relative difference in excess deaths observed across different ethnic groups.

## Figures and Tables

**Table 1:** List of SARS-CoV-2 receptor and co-receptor SNP associations in other disease(s) and proposed mechanism(s).

Variant ID	Mutation	Disease(s)	Genetic Ancestry	Proposed Mechanism	Citation	MAF
ACE2						
rs1978124	T>C	Cardiovascular disease	Uygurs—East Asian	Intron variant	[27]	AFR: 0.90
						EUR: 0.53
						SAS: 0.78
rs233575	G>A	Cardiovascular disease, Hypertension	East Asian and European	Intron variant	[27,28]	AFR: 0.99
						EUR: 0.65
						SAS: 0.82
rs1514282	T>C	Blood pressure	East Asian	Intron variant	[29]	AFR: 0.32
						EUR: 0.00
						SAS: 0.09
rs16997078	A>G	No disease associations found		Intron Variant		AFR: 0.24
						EUR: 0.00
						SAS: 0.09
rs2097723	T>C	Blood pressure	East Asian	Intron Variant	[30]	AFR: 0.07
						EUR: 0.28
						SAS: 0.22
rs879922	C>G	Hypertension, Cardiovascular disease, blood pressure and T2D.	East Asian	Intron Variant	[27,31]	AFR: 0.45
						EUR: 0.65
						SAS: 0.71
rs2106809	A>G	Blood pressure	European	Intron Variant	[32]	AFR: 0.09
						EUR: 0.25
						SAS: 0.48
rs2048683	T>G	Cardiovascular disease	East Asian	Intron Variant	[27]	AFR: 0.81
						EUR: 0.65
						SAS: 0.80
rs4646120	G>A	Shared haplotype between South Asian and East Eurasians for host susceptibility to SARS-CoV-2.	South Asian	Intron Variant	[33]	AFR: 0.68
						EUR: 0.53
						SAS: 0.78
rs4646140	C>T	Blood pressure	East Asian	Intron Variant	[31]	AFR: 0.13
						EUR: 0.00
						SAS: 0.09
rs35697037	G>A	Significant correlation with dizziness symptoms in mild traumatic brain injury.	East Asian	Intron Variant	[34]	AFR: 0.27
						EUR: 0.38
						SAS: 0.24
rs714205	C>G	Hypertension, and Retinopathy in T2D	East Asian	Intron Variant	[29,35]	AFR: 0.10
						EUR: 0.21
						SAS: 0.47
TMPRSS2						
rs463727	T>A	Susceptibility and severity of respiratory disease	East Asian And European	500 KB downstream variant	[36]	AFR: 0.08
						EUR: 0.46
						SAS: 0.34
rs35041537	C>T	Infectivity and Progression of SARS-CoV-2	South Asian	Intron Variant	[37]	AFR: 0.11
						EUR: 0.46
						SAS: 0.34
rs7275220	G>A	Breast Cancer	European	Intron Variant	[38]	AFR: 0.49
						EUR: 0.75
						SAS: 0.66
rs456298	T>A	Associated with high risk of SARS-CoV-2	Mexican -European	3 Prime UTR Variant	[39]	AFR: 0.63
						EUR: 0.83
						SAS: 0.68
rs2070788	G>A	Susceptibility to infectious disease	East Asian	Intron Variant	[40]	AFR: 0.73
						EUR: 0.54
						SAS: 0.53
rs8127290	G>A	OAS variant associated with rubella	European	None	[41]	AFR: 0.35
						EUR: 0.16
						SAS: 0.22
rs383510	T>C	Susceptibility to infectious disease	East Asian	Intron Variant	[40]	AFR: 0.67
						EUR: 0.51
						SAS: 0.55
rs11910678	T>C	Susceptibility to SARS-CoV-2	None	3 Prime UTR Variant	[42]	AFR: 0.14
						EUR: 0.00
						SAS: 0.003
rs1557372	C>T	Alzheimer’s disease	East Asian	None	[43]	AFR: 0.50
						EUR: 0.37
						SAS: 0.32
rs75603675	C>A	Associated with SARS-CoV-2 disease susceptibility and severity	Eastern European	Missense Variant	[44]	AFR: 0.30
						EUR: 0.40
						SAS: 0.22
NRP1						
rs2804495	G>T	Neovascular age-related macular degeneration	European	Intron variant	[45]	AFR: 0.24
						EUR: 0.71
						SAS: 0.57
rs927099	T>C	Late-onset Alzheimer disease.	European	Intron Variant	[46]	AFR: 0.88
						EUR: 0.50
						SAS: 0.55
rs1048804	A>G	Type 1 diabetes	European	Synonymous Variant	[47]	AFR: 0.57
						EUR: 0.25
						SAS: 0.37
rs10080	G>A	Risk of tetralogy of Fallot	East Asian	Non-Coding Transcript Variant	[48]	AFR: 0.70
						EUR: 0.43
						SAS: 0.50
rs1319013	T>G	Late-onset Alzheimer disease	European	Intron variant	[49]	AFR: 0.37
						EUR: 0.53
						SAS: 0.52
rs1571781	A>G	Late-onset Alzheimer disease	European	Intron variant	[46]	AFR: 0.78
						EUR: 0.62
						SAS: 0.51
rs2070296	C>T	Associated with worse response to ranibizumab treatment in neovascular age-related macular degeneration	European	Synonymous Variant	[45]	AFR: 0.32
						EUR: 0.16
						SAS: 0.23
rs2506144	C>T	No disease associations	European	Non-Coding Transcript Variant		AFR: 0.33
						EUR: 0.18
						SAS: 0.09
rs1888686	T>C	Late-onset Alzheimer disease	European	Intron Variant	[46]	AFR: 0.34
						EUR: 0.21
						SAS: 0.26
rs12573218	C>T	Osteonecrosis of the femoral head	East Asian	Intron Variant	[50]	AFR: 0.02
						EUR: 0.15
						SAS: 0.10
rs1010826	G>A	Type 1 diabetes	European	Intron Variant	[47]	AFR: 0.35
						EUR: 0.23
						SAS: 0.41
rs1331326	C>T	Late-onset Alzheimer disease	European	Intron Variant	[46]	AFR: 0.53
						EUR: 0.65
						SAS: 0.65
rs1888685	C>T	Late-onset Alzheimer disease	European	Intron Variant	[46]	AFR: 0.01
						EUR: 0.13
						SAS: 0.23
rs12358370	C>G	Osteonecrosis of the femoral head	East Asian	Intron Variant	[50]	AFR: 0.03
						EUR: 0.15
						SAS: 0.12
rs2228638	C>T	Risk of tetralogy of Fallot	East Asian	Missense Variant	[48]	AFR: 0.01
						EUR: 0.11
						SAS: 0.13
CD147						
rs8637	A>G	Coronary Heart Disease	East Asian	3 Prime UTR Variant	[51]	AFR: 0.97
						EUR: 0.47
						SAS: 0.83
rs8259	T>A	Coronary Heart Disease, A miRNA-492 binding-site polymorphism in BSG (basigin) confers risk to psoriasis in central south Chinese population.	East Asian	3 Prime UTR Variant	[51,52]	AFR: 0.56
						EUR: 0.30
						SAS: 0.63
rs4919862	T>C	Carotid Plaque Risk in Acute Cerebral Infarction	East Asian	Intron Variant	[53]	AFR: 0.98
						EUR: 0.75
						SAS: 0.92
rs4919859	G>C	Coronary Heart Disease	East Asian	Intron Variant, 2 KB Upstream Variant	[51]	AFR: 0.51
						EUR: 0.35
						SAS: 0.50
rs6758	G>A	Coronary Heart Disease, Hyperpolarization-activated cyclic nucleotide-gated channels and its relationship with neuroticism, cognition, and risk of depression	East Asian and European	3 Prime UTR Variant	[51,54]	AFR: 0.21
						EUR: 0.09
						SAS: 0.25

## Data Availability

Not applicable.

## References

[1] Patel R., Kaki M., Potluri V.S., Kahar P., Khanna D. (2022). A comprehensive review of SARS-CoV-2 vaccines: Pfizer, Moderna & Johnson & Johnson. Hum. Vaccines Immunother..

[2] Rodriguez E.V., Bouazza F.-Z., Dauby N., Mullier F., D’otreppe S., Jissendi Tchofo P., Bartiaux M., Sirjacques C., Roman A., Hermans C. (2022). Fatal vaccine-induced immune thrombotic thrombocytopenia (VITT) post Ad26. COV2. S: First documented case outside US. Infection.

[3] Wang C., Liu B., Zhang S., Huang N., Zhao T., Lu Q.B., Cui F. (2023). Differences in incidence and fatality of COVID-19 by SARS-CoV-2 Omicron variant versus Delta variant in relation to vaccine coverage: A world-wide review. J. Med. Virol..

[4] Samson M., Libert F., Doranz B.J., Rucker J., Liesnard C., Farber C.-M., Saragosti S., Lapouméroulie C., Cognaux J., Forceille C. (1996). Resistance to HIV-1 infection in caucasian individuals bearing mutant alleles of the CCR-5 chemokine receptor gene. Nature.

[5] De Silva E., Stumpf M.P.H. (2004). HIV and the CCR5-Δ32 resistance allele. FEMS Microbiol. Lett..

[6] Martinson J.J., Chapman N.H., Rees D.C., Liu Y.-T., Clegg J.B. (1997). Global distribution of the CCR5 gene 32-basepair deletion. Nat. Genet..

[7] Lederman M.M., Pike E. (2017). Ten Years HIV Free: An Interview with “The Berlin Patient,” Timothy Ray Brown. Pathog. Immun..

[8] Brown T.R. (2015). I am the Berlin patient: A personal reflection. AIDS Res. Hum. Retrovir..

[9] Gupta R.K., Peppa D., Hill A.L., Gálvez C., Salgado M., Pace M., Mccoy L.E., Griffith S.A., Thornhill J., Alrubayyi A. (2020). Evidence for HIV-1 cure after CCR5Δ32/Δ32 allogeneic haemopoietic stem-cell transplantation 30 months post analytical treatment interruption: A case report. Lancet HIV.

[10] Li H., Liu Z., Han Q., Li Y., Chen J. (2006). Association of genetic polymorphism of low-density lipoprotein receptor with chronic viral hepatitis C infection in Han Chinese. J. Med. Virol..

[11] Harada R., Kimura M., Sato Y., Taniguchi T., Tomonari T., Tanaka T., Tanaka H., Muguruma N., Shinomiya H., Honda H. (2018). APOB codon 4311 polymorphism is associated with hepatitis C virus infection through altered lipid metabolism. BMC Gastroenterol..

[12] Naga M., Amin M., Algendy D., Elbadry A., Fawzi M., Foda A., Esmat S., Sabry D., Rashed L., Gabal S. (2015). Low-density lipoprotein receptor genetic polymorphism in chronic hepatitis C virus Egyptian patients affects treatment response. World J. Gastroenterol..

[13] Hu H.H., Liu J., Lin Y.L., Luo W.S., Chu Y.J., Chang C.L., Jen C.L., Lee M.H., Lu S.N., Wang L.Y. (2016). The rs2296651 (S267F) variant on NTCP (SLC10A1) is inversely associated with chronic hepatitis B and progression to cirrhosis and hepatocellular carcinoma in patients with chronic hepatitis B. Gut.

[14] Chuaypen N., Tuyapala N., Pinjaroen N., Payungporn S., Tangkijvanich P. (2019). Association of NTCP polymorphisms with clinical outcome of hepatitis B infection in Thai individuals. BMC Med. Genet..

[15] Hoffmann M., Kleine-Weber H., Schroeder S., Krüger N., Herrler T., Erichsen S., Schiergens T.S., Herrler G., Wu N.H., Nitsche A. (2020). SARS-CoV-2 Cell Entry Depends on ACE2 and TMPRSS2 and Is Blocked by a Clinically Proven Protease Inhibitor. Cell.

[16] Mayi B.S., Leibowitz J.A., Woods A.T., Ammon K.A., Liu A.E., Raja A. (2021). The role of Neuropilin-1 in COVID-19. PLoS Pathog..

[17] Wang K., Chen W., Zhang Z., Deng Y., Lian J.-Q., Du P., Wei D., Zhang Y., Sun X.-X., Gong L. (2020). CD147-spike protein is a novel route for SARS-CoV-2 infection to host cells. Signal Transduct. Target. Ther..

[18] Zhang Q., Chen C.Z., Swaroop M., Xu M., Wang L., Lee J., Wang A.Q., Pradhan M., Hagen N., Chen L. (2020). Heparan sulfate assists SARS-CoV-2 in cell entry and can be targeted by approved drugs in vitro. Cell Discov..

[19] Vahidy F.S., Nicolas J.C., Meeks J.R., Khan O., Pan A., Jones S.L., Masud F., Sostman H.D., Phillips R., Andrieni J.D. (2020). Racial and ethnic disparities in SARS-CoV-2 pandemic: Analysis of a COVID-19 observational registry for a diverse US metropolitan population. BMJ Open.

[20] Delshad M., Sanaei M.J., Pourbagheri-Sigaroodi A., Bashash D. (2022). Host genetic diversity and genetic variations of SARS-CoV-2 in COVID-19 pathogenesis and the effectiveness of vaccination. Int. Immunopharmacol..

[21] Bwire G., Ario A.R., Eyu P., Ocom F., Wamala J.F., Kusi K.A., Ndeketa L., Jambo K.C., Wanyenze R.K., Talisuna A.O. (2022). The COVID-19 pandemic in the African continent. BMC Med..

[22] WHO (2021). WHO Coronavirus (COVID-19) Dashboard|WHO Coronavirus (COVID-19) Dashboard with Vaccination Data.

[23] European Centre for Disease Prevention and Control (CDC) (2020). European Centre for Disease Prevention and Control (CDC) COVID-19 Situation Update Worldwide, as of 30 June 2020.

[24] Chitungo I., Dzobo M., Hlongwa M., Dzinamarira T. (2020). COVID-19: Unpacking the low number of cases in Africa. Public Health Pract..

[25] Kircheis R., Schuster M., Planz O. (2021). COVID-19: Mechanistic Model of the African Paradox Supports the Central Role of the NF-κB Pathway. Viruses.

[26] Adams J., Mackenzie M.J., Amegah A.K., Ezeh A., Gadanya M.A., Omigbodun A., Sarki A.M., Thistle P., Ziraba A.K., Stranges S. (2021). The conundrum of low COVID-19 mortality burden in sub-Saharan Africa: Myth or reality?. Glob. Health Sci. Pract..

[27] Liu C., Li Y., Guan T., Lai Y., Shen Y., Zeyaweiding A., Zhao H., Li F., Maimaiti T. (2018). ACE2 polymorphisms associated with cardiovascular risk in Uygurs with type 2 diabetes mellitus. Cardiovasc. Diabetol..

[28] Lieb W., Graf J., Götz A., König I.R., Mayer B., Fischer M., Stritzke J., Hengstenberg C., Holmer S.R., Döring A. (2006). Association of angiotensin-converting enzyme 2 (ACE2) gene polymorphisms with parameters of left ventricular hypertrophy in men. Results of the MONICA Augsburg echocardiographic substudy. J. Mol. Med..

[29] Zhao Q., Hixson J.E., Rao D.C., Gu D., Jaquish C.E., Rice T., Shimmin L.C., Chen J., Cao J., Kelly T.N. (2010). Genetic variants in the apelin system and blood pressure responses to dietary sodium interventions: A family-based association study. J. Hypertens..

[30] Fan X.H., Wang Y.B., Wang H., Sun K., Zhang W.L., Song X.D., Cheng J.Z., Wu H.Y., Zhou X.L., Hui R.T. (2009). Polymorphisms of angiotensin-converting enzyme (ACE) and ACE2 are not associated with orthostatic blood pressure dysregulation in hypertensive patients. Acta Pharmacol. Sin..

[31] Zhao Q., Gu D., Kelly T.N., Hixson J.E., Rao D.C., Jaquish C.E., Chen J., Huang J., Chen C.S., Gu C.C. (2010). Association of genetic variants in the apelin-APJ system and ACE2 with blood pressure responses to potassium supplementation: The GenSalt study. Am. J. Hypertens..

[32] Lozano-Gonzalez K., Padilla-Rodríguez E., Texis T., Gutiérrez M.N., Rodríguez-Dorantes M., Cuevas-Córdoba B., Ramírez-García E., Mino-León D., Sánchez-García S., Gonzalez-Covarrubias V. (2020). Allele Frequency of ACE2 Intron Variants and Its Association with Blood Pressure. DNA Cell Biol..

[33] Srivastava A., Pandey R.K., Singh P.P., Kumar P., Rasalkar A.A., Tamang R., van Driem G., Shrivastava P., Chaubey G. (2020). Most frequent South Asian haplotypes of ACE2 share identity by descent with East Eurasian populations. PLoS ONE.

[34] Wang Y.-J., Hsu Y.-W., Chang C.-M., Wu C.-C., Ou J.-C., Tsai Y.-R., Chiu W.-T., Chang W.-C., Chiang Y.-H., Chen K.-Y. (2014). The influence of BMX gene polymorphisms on clinical symptoms after mild traumatic brain injury. BioMed Res. Int..

[35] Meng N., Zhang Y., Ma J., Li H., Zhou F., Qu Y. (2015). Association of polymorphisms of angiotensin I converting enzyme 2 with retinopathy in type 2 diabetes mellitus among Chinese individuals. Eye.

[36] Ghafouri-Fard S., Noroozi R., Vafaee R., Branicki W., Poṡpiech E., Pyrc K., Łabaj P.P., Omrani M.D., Taheri M., Sanak M. (2020). Effects of host genetic variations on response to, susceptibility and severity of respiratory infections. Biomed. Pharmacother..

[37] Iyer G.R., Samajder S., Zubeda S., Devi S.N.S., Mali V., Pv S.K., Sharma A., Abbas N.Z., Bora N.S., Narravula A. (2020). Infectivity and Progression of COVID-19 Based on Selected Host Candidate Gene Variants. Front. Genet..

[38] Luostari K., Hartikainen J.M., Tengström M., Palvimo J.J., Kataja V., Mannermaa A., Kosma V.M. (2014). Type II transmembrane serine protease gene variants associate with breast cancer. PLoS ONE.

[39] Posadas-Sánchez R., Fragoso J.M., Sánchez-Muñoz F., Rojas-Velasco G., Ramírez-Bello J., López-Reyes A., Martínez-Gómez L.E., Sierra-Fernández C., Rodríguez-Reyna T., Regino-Zamarripa N.E. (2020). Association of the Transmembrane Serine Protease-2 (TMPRSS2) Polymorphisms with COVID-19. Viruses.

[40] Cheng Z., Zhou J., To K.K., Chu H., Li C., Wang D., Yang D., Zheng S., Hao K., Bossé Y. (2015). Identification of TMPRSS2 as a Susceptibility Gene for Severe 2009 Pandemic A(H1N1) Influenza and A(H7N9) Influenza. J. Infect. Dis..

[41] Haralambieva I.H., Dhiman N., Ovsyannikova I.G., Vierkant R.A., Pankratz V.S., Jacobson R.M., Poland G.A. (2010). 2′-5′-Oligoadenylate synthetase single-nucleotide polymorphisms and haplotypes are associated with variations in immune responses to rubella vaccine. Hum. Immunol..

[42] Senapati S., Kumar S., Singh A.K., Banerjee P., Bhagavatula S. (2020). Assessment of risk conferred by coding and regulatory variations of TMPRSS2 and CD26 in susceptibility to SARS-CoV-2 infection in human. J. Genet..

[43] Ma S.L., Huang W., Tang N.L., Lam L.C. (2012). MxA polymorphisms are associated with risk and age-at-onset in Alzheimer disease and accelerated cognitive decline in Chinese elders. Rejuvenation Res..

[44] Minashkin M.M., Grigortsevich N.Y., Kamaeva A.S., Barzanova V.V., Traspov A.A., Godkov M.A., Ageev F.A., Petrikov S.S., Pozdnyakova N.V. (2022). The Role of Genetic Factors in the Development of Acute Respiratory Viral Infection COVID-19: Predicting Severe Course and Outcomes. Biomedicines.

[45] Lorés-Motta L., van Asten F., Muether P.S., Smailhodzic D., Groenewoud J.M., Omar A., Chen J., Koenekoop R.K., Fauser S., Hoyng C.B. (2016). A genetic variant in NRP1 is associated with worse response to ranibizumab treatment in neovascular age-related macular degeneration. Pharm. Genom..

[46] Grupe A., Li Y., Rowland C., Nowotny P., Hinrichs A.L., Smemo S., Kauwe J.S., Maxwell T.J., Cherny S., Doil L. (2006). A scan of chromosome 10 identifies a novel locus showing strong association with late-onset Alzheimer disease. Am. J. Hum. Genet..

[47] Nejentsev S., Smink L.J., Smyth D., Bailey R., Lowe C.E., Payne F., Masters J., Godfrey L., Lam A., Burren O. (2007). Sequencing and association analysis of the type 1 diabetes-linked region on chromosome 10p12-q11. BMC Genet..

[48] Fan S.H., Shen Z.Y., Xiao Y.M. (2018). Functional polymorphisms of the neuropilin 1 gene are associated with the risk of tetralogy of Fallot in a Chinese Han population. Gene.

[49] Thornton-Wells T.A., Moore J.H., Martin E.R., Pericak-Vance M.A., Haines J.L. (2008). Confronting complexity in late-onset Alzheimer disease: Application of two-stage analysis approach addressing heterogeneity and epistasis. Genet. Epidemiol..

[50] Hong J.M., Kim T.H., Kim H.J., Park E.K., Yang E.K., Kim S.Y. (2010). Genetic association of angiogenesis- and hypoxia-related gene polymorphisms with osteonecrosis of the femoral head. Exp. Mol. Med..

[51] Weng Y., Chen T., Ren J., Lu D., Liu X., Lin S., Xu C., Lou J., Chen X., Tang L. (2020). The Association Between Extracellular Matrix Metalloproteinase Inducer Polymorphisms and Coronary Heart Disease: A Potential Way to Predict Disease. DNA Cell Biol..

[52] Wu L.-S., Li F.-F., Sun L.-D., Li D., Su J., Kuang Y.-H., Chen G., Chen X.-P., Chen X. (2011). A miRNA-492 binding-site polymorphism in BSG (basigin) confers risk to psoriasis in central south Chinese population. Hum. Genet..

[53] Jin W., Wu W., Yang K., Shen F., Fu N., Feng Y., Fu Y. (2020). The Single Nucleotide Polymorphisms of Chromosome 9p21 and CD147 Were Relevant with the Carotid Plaque Risk in Acute Cerebral Infarction Patients Among Chinese Han Population. J. Mol. Neurosci..

[54] Mcintosh A.M., Simen A.A., Evans K.L., Hall J., MacIntyre D.J., Blackwood D., Morris A.D., Smith B.H., Dominiczak A., Porteous D. (2012). Genetic variation in Hyperpolarization-activated cyclic nucleotide-gated channels and its relationship with neuroticism, cognition and risk of depression. Front. Genet..

[55] Campbell M.C., Tishkoff S.A. (2008). African genetic diversity: Implications for human demographic history, modern human origins, and complex disease mapping. Annu. Rev. Genomics. Hum. Genet..

[56] Yu N., Chen F.-C., Ota S., Jorde L.B., Pamilo P., Patthy L., Ramsay M., Jenkins T., Shyue S.-K., Li W.-H. (2002). Larger Genetic Differences Within Africans Than Between Africans and Eurasians. Genetics.

[57] Tucci S., Akey J.M. (2019). The long walk to African genomics. Genome Biol..

[58] Choudhury A., Aron S., Botigué L.R., Sengupta D., Botha G., Bensellak T., Wells G., Kumuthini J., Shriner D., Fakim Y.J. (2020). High-depth African genomes inform human migration and health. Nature.

[59] Acosta E. (2022). Global estimates of excess deaths from COVID-19. Nature.

[60] Msemburi W., Karlinsky A., Knutson V., Aleshin-Guendel S., Chatterji S., Wakefield J. (2022). The WHO estimates of excess mortality associated with the COVID-19 pandemic. Nature.

[61] Wang H., Paulson K.R., Pease S.A., Watson S., Comfort H., Zheng P., Aravkin A.Y., Bisignano C., Barber R.M., Alam T. (2022). Estimating excess mortality due to the COVID-19 pandemic: A systematic analysis of COVID-19-related mortality, 2020–2021. Lancet.

[62] Wachira L.J., Arena R., Sallis J.F., Lambert E.V., Ong’Wen O.M., Laddu D.R., Onywera V., Oyeyemi A.L. (2022). Why are COVID-19 effects less severe in Sub-Saharan Africa? Moving more and sitting less may be a primary reason. Prog. Cardiovasc. Dis..

[63] Wamai R.G., Hirsch J.L., van Damme W., Alnwick D., Bailey R.C., Hodgins S., Alam U., Anyona M. (2021). What Could Explain the Lower COVID-19 Burden in Africa despite Considerable Circulation of the SARS-CoV-2 Virus?. Int. J. Environ. Res. Public Health.

[64] Nei M. (1995). Genetic support for the out-of-Africa theory of human evolution. Proc. Natl. Acad. Sci. USA.

[65] Donoghue M., Hsieh F., Baronas E., Godbout K., Gosselin M., Stagliano N., Donovan M., Woolf B., Robison K., Jeyaseelan R. (2000). A novel angiotensin-converting enzyme–related carboxypeptidase (ACE2) converts angiotensin I to angiotensin 1–9. Circ. Res..

[66] Patel S., Rauf A., Khan H., Abu-Izneid T. (2017). Renin-angiotensin-aldosterone (RAAS): The ubiquitous system for homeostasis and pathologies. Biomed. Pharmacother..

[67] Santos RA S., Sampaio W.O., Alzamora A.C., Motta-Santos D., Alenina N., Bader M., Campagnole-Santos M.J. (2017). The ACE2/angiotensin-(1–7)/MAS axis of the renin-angiotensin system: Focus on angiotensin-(1–7). Physiol. Rev..

[68] Hamming I., Timens W., Bulthuis M., Lely A., Navis G.V., van Goor H. (2004). Tissue distribution of ACE2 protein, the functional receptor for SARS coronavirus. A first step in understanding SARS pathogenesis. J. Pathol. A J. Pathol. Soc. Great Br. Irel..

[69] Zou X., Chen K., Zou J., Han P., Hao J., Han Z. (2020). Single-cell RNA-seq data analysis on the receptor ACE2 expression reveals the potential risk of different human organs vulnerable to 2019-nCoV infection. Front. Med..

[70] Gu J., Korteweg C. (2007). Pathology and pathogenesis of severe acute respiratory syndrome. Am. J. Pathol..

[71] Gu J., Gong E., Zhang B., Zheng J., Gao Z., Zhong Y., Zou W., Zhan J., Wang S., Xie Z. (2005). Multiple organ infection and the pathogenesis of SARS. J. Exp. Med..

[72] Beacon T.H., Delcuve G.P., Davie J.R. (2021). Epigenetic regulation of ACE2, the receptor of the SARS-CoV-2 virus1. Genome.

[73] Patel S.K., Velkoska E., Freeman M., Wai B., Lancefield T.F., Burrell L.M. (2014). From gene to protein—Experimental and clinical studies of ACE2 in blood pressure control and arterial hypertension. Front. Physiol..

[74] Scialo F., Daniele A., Amato F., Pastore L., Matera M.G., Cazzola M., Castaldo G., Bianco A. (2020). ACE2: The major cell entry receptor for SARS-CoV-2. Lung.

[75] Jackson C.B., Farzan M., Chen B., Choe H. (2022). Mechanisms of SARS-CoV-2 entry into cells. Nat. Rev. Mol. Cell Biol..

[76] Samavati L., Uhal B.D. (2020). ACE2, much more than just a receptor for SARS-CoV-2. Front. Cell. Infect. Microbiol..

[77] Turner A.J., Tipnis S.R., Guy J.L., Rice G.I., Hooper N.M. (2002). ACEH/ACE2 is a novel mammalian metallocarboxypeptidase and a homologue of angiotensin-converting enzyme insensitive to ACE inhibitors. Can. J. Physiol. Pharmacol..

[78] Li W., Moore M.J., Vasilieva N., Sui J., Wong S.K., Berne M.A., Somasundaran M., Sullivan J.L., Luzuriaga K., Greenough T.C. (2003). Angiotensin-converting enzyme 2 is a functional receptor for the SARS coronavirus. Nature.

[79] Yan R., Zhang Y., Li Y., Xia L., Guo Y., Zhou Q. (2020). Structural basis for the recognition of SARS-CoV-2 by full-length human ACE2. Science.

[80] Zhou P., Yang X.-L., Wang X.-G., Hu B., Zhang L., Zhang W., Si H.-R., Zhu Y., Li B., Huang C.-L. (2020). A pneumonia outbreak associated with a new coronavirus of probable bat origin. Nature.

[81] Li F., Li W., Farzan M., Harrison S.C. (2005). Structure of SARS coronavirus spike receptor-binding domain complexed with receptor. Science.

[82] Li W., Zhang C., Sui J., Kuhn J.H., Moore M.J., Luo S., Wong S.-K., Huang I.-C., Xu K., Vasilieva N. (2005). Receptor and viral determinants of SARS-coronavirus adaptation to human ACE2. EMBO J..

[83] Dhangadamajhi G., Mohapatra B.N., Kar S.K., Ranjit M. (2010). Gene polymorphisms in angiotensin I converting enzyme (ACE I/D) and angiotensin II converting enzyme (ACE2 C→T) protect against cerebral malaria in Indian adults. Infect. Genet. Evol..

[84] Rigat B., Hubert C., Alhenc-Gelas F., Cambien F., Corvol P., Soubrier F. (1990). An insertion/deletion polymorphism in the angiotensin I-converting enzyme gene accounting for half the variance of serum enzyme levels. J. Clin. Investig..

[85] Gallego-Delgado J., Walther T., Rodriguez A. (2016). The high blood pressure-malaria protection hypothesis. Circ. Res..

[86] Sampson U.K., Edwards T.L., Jahangir E., Munro H., Wariboko M., Wassef M.G., Fazio S., Mensah G.A., Kabagambe E.K., Blot W.J. (2014). Factors associated with the prevalence of hypertension in the southeastern United States: Insights from 69 211 blacks and whites in the southern community cohort study. Circ. Cardiovasc. Qual. Outcomes.

[87] Riordan J.F. (2003). Angiotensin-I-converting enzyme and its relatives. Genome Biol..

[88] Rusmini M., Uva P., Amoroso A., Tolomeo M., Cavalli A. (2021). How Genetics Might Explain the Unusual Link Between Malaria and COVID-19. Front. Med..

[89] Pouladi N., Abdolahi S. (2021). Investigating the ACE2 polymorphisms in COVID-19 susceptibility: An in silico analysis. Mol. Genet. Genom. Med..

[90] De A., Tiwari A., Dash M., Sinha A. (2021). ACE2 mutation might explain lower COVID-19 burden in malaria endemic areas. Hum. Cell.

[91] Hussein M.I.H., Albashir A.A.D., Elawad O.A.M.A., Homeida A. (2020). Malaria and COVID-19: Unmasking their ties. Malar. J..

[92] Badraoui R., Adnan M., Bardakci F., Alreshidi M.M. (2021). Chloroquine and hydroxychloroquine interact differently with ACE2 domains reported to bind with the coronavirus spike protein: Mediation by ACE2 polymorphism. Molecules.

[93] Acquah S. (2020). Implications of COVID-19 Pandemic on Evolution of Diabetes in Malaria-Endemic African Region. J. Diabetes Res..

[94] Möhlendick B., Schönfelder K., Breuckmann K., Elsner C., Babel N., Balfanz P., Dahl E., Dreher M., Fistera D., Herbstreit F. (2021). ACE2 polymorphism and susceptibility for SARS-CoV-2 infection and severity of COVID-19. Pharm. Genom..

[95] Wu Y.H., Li J.Y., Wang C., Zhang L.M., Qiao H. (2017). The ACE 2 G8790A polymorphism: Involvement in type 2 diabetes mellitus combined with cerebral stroke. J. Clin. Lab. Anal..

[96] Sabater Molina M., Nicolás Rocamora E., Bendicho A.I., Vázquez E.G., Zorio E., Rodriguez F.D., Gil Ortuño C., Rodríguez A.I., Sánchez-López A.J., Jara Rubio R. (2022). Polymorphisms in ACE, ACE2, AGTR1 genes and severity of COVID-19 disease. PLoS ONE.

[97] Alimoradi N., Sharqi M., Firouzabadi D., Sadeghi M.M., Moezzi M.I., Firouzabadi N. (2022). SNPs of ACE1 (rs4343) and ACE2 (rs2285666) genes are linked to SARS-CoV-2 infection but not with the severity of disease. Virol. J..

[98] Karakaş Çelik S., Çakmak Genç G., Pişkin N., Açikgöz B., Altinsoy B., Kurucu Işsiz B., Dursun A. (2021). Polymorphisms of ACE (I/D) and ACE2 receptor gene (Rs2106809, Rs2285666) are not related to the clinical course of COVID-19: A case study. J. Med. Virol..

[99] Abdelsattar S., Kasemy Z.A., Ewida S.F., Abo-Elsoud R.A.A., Zytoon A.A., Abdelaal G.A., Abdelgawad A.S., Khalil F.O., Kamel H. (2022). ACE2 and TMPRSS2 SNPs as Determinants of Susceptibility to, and Severity of, a COVID-19 Infection. Br. J. Biomed. Sci..

[100] Patel S.K., Wai B., Ord M., MacIsaac R.J., Grant S., Velkoska E., Panagiotopoulos S., Jerums G., Srivastava P., Burrell L.M. (2012). Association of ACE2 genetic variants with blood pressure, left ventricular mass, and cardiac function in Caucasians with type 2 diabetes. Am. J. Hypertens..

[101] Pan Y., Wang T., Li Y., Guan T., Lai Y., Shen Y., Zeyaweiding A., Maimaiti T., Li F., Zhao H. (2018). Association of ACE2 polymorphisms with susceptibility to essential hypertension and dyslipidemia in Xinjiang, China. Lipids Health Dis..

[102] Hamet P., Pausova Z., Attaoua R., Hishmih C., Haloui M., Shin J., Paus T., Abrahamowicz M., Gaudet D., Santucci L. (2021). SARS-CoV-2 Receptor ACE2 Gene Is Associated with Hypertension and Severity of COVID 19: Interaction with Sex, Obesity, and Smoking. Am. J. Hypertens..

[103] Faridzadeh A., Mahmoudi M., Ghaffarpour S., Zamani M.S., Hoseinzadeh A., Naghizadeh M.M., Ghazanfari T. (2022). The role of ACE1 I/D and ACE2 polymorphism in the outcome of Iranian COVID-19 patients: A case-control study. Front. Genet..

[104] Saniasiaya J., Kulasegarah J. (2021). Dizziness and COVID-19. Ear Nose Throat J..

[105] Korres G., Kitsos D.K., Kaski D., Tsogka A., Giannopoulos S., Giannopapas V., Sideris G., Tyrellis G., Voumvourakis K. (2022). The Prevalence of Dizziness and Vertigo in COVID-19 Patients: A Systematic Review. Brain Sci..

[106] Jafari Z., Kolb B.E., Mohajerani M.H. (2022). Hearing loss, tinnitus, and dizziness in COVID-19: A systematic review and meta-analysis. Can. J. Neurol. Sci..

[107] Han W., Quan B., Guo Y., Zhang J., Lu Y., Feng G., Wu Q., Fang F., Cheng L., Jiao N. (2020). The course of clinical diagnosis and treatment of a case infected with coronavirus disease 2019. J. Med. Virol..

[108] Liu C., Zhou J., Xia L., Cheng X., Lu D. (2020). 18F-FDG PET/CT and serial chest CT findings in a COVID-19 patient with dynamic clinical characteristics in different period. Clin. Nucl. Med..

[109] Chen J., Peng S., Zhang B., Liu Z., Liu L., Zhang W. (2020). An uncommon manifestation of COVID-19 pneumonia on CT scan with small cavities in the lungs: A case report. Medicine.

[110] Wander P.L., Lowy E., Beste L.A., Tulloch-Palomino L., Korpak A., Peterson A.C., Kahn S.E., Boyko E.J. (2022). The incidence of diabetes among 2,777,768 veterans with and without recent SARS-CoV-2 infection. Diabetes Care.

[111] Jalaleddine N., Bouzid A., Hachim M., Sharif-Askari N.S., Mahboub B., Senok A., Halwani R., Hamoudi R.A., Al Heialy S. (2022). ACE2 polymorphisms impact COVID-19 severity in obese patients. Sci. Rep..

[112] Elbadri S.A., Abdallah N.M.A., El-Shokry M., Gaber A., Elsayed M.K. (2022). Association between single nucleotide polymorphism of human angiotensin-converting enzyme 2 gene locus and clinical severity of COVID-19. Egypt. J. Med. Hum. Genet..

[113] He Y., Yang W., Liu S., Gan L., Zhang F., Mu C., Wang J., Qu L., Wang R., Deng J. (2017). Interactions between angiotensin-converting enzyme-2 polymorphisms and high salt intake increase the risk of hypertension in the Chinese Wa population. Int. J. Clin. Exp. Pathol..

[114] Darbani B. (2020). The expression and polymorphism of entry machinery for COVID-19 in human: Juxtaposing population groups, gender, and different tissues. Int. J. Environ. Res. Public Health.

[115] Khayat A.S., de Assumpção P.P., Meireles Khayat B.C., Thomaz Araújo T.M., Batista-Gomes J.A., Imbiriba L.C., Ishak G., de Assumpção P.B., Moreira F.C., Burbano R.R. (2020). ACE2 polymorphisms as potential players in COVID-19 outcome. PLoS ONE.

[116] Gemmati D., Tisato V. (2020). Genetic Hypothesis and Pharmacogenetics Side of Renin-Angiotensin-System in COVID-19. Genes.

[117] Paniri A., Hosseini M.M., Moballegh-Eslam M., Akhavan-Niaki H. (2021). Comprehensive in silico identification of impacts of ACE2 SNPs on COVID-19 susceptibility in different populations. Gene Rep..

[118] Cao Y., Li L., Feng Z., Wan S., Huang P., Sun X., Wen F., Huang X., Ning G., Wang W. (2020). Comparative genetic analysis of the novel coronavirus (2019-nCoV/SARS-CoV-2) receptor ACE2 in different populations. Cell Discov..

[119] Guo X., Chen Z., Xia Y., Lin W., Li H. (2020). Investigation of the genetic variation in ACE2 on the structural recognition by the novel coronavirus (SARS-CoV-2). J. Transl. Med..

[120] Abe M., Tahara M., Sakai K., Yamaguchi H., Kanou K., Shirato K., Kawase M., Noda M., Kimura H., Matsuyama S. (2013). TMPRSS2 is an activating protease for respiratory parainfluenza viruses. J. Virol..

[121] Glowacka I., Bertram S., Müller M.A., Allen P., Soilleux E., Pfefferle S., Steffen I., Tsegaye T.S., He Y., Gnirss K. (2011). Evidence that TMPRSS2 activates the severe acute respiratory syndrome coronavirus spike protein for membrane fusion and reduces viral control by the humoral immune response. J. Virol..

[122] Bertram S., Dijkman R., Habjan M., Heurich A., Gierer S., Glowacka I., Welsch K., Winkler M., Schneider H., Hofmann-Winkler H. (2013). TMPRSS2 activates the human coronavirus 229E for cathepsin-independent host cell entry and is expressed in viral target cells in the respiratory epithelium. J. Virol..

[123] Shirato K., Kawase M., Matsuyama S. (2013). Middle East respiratory syndrome coronavirus infection mediated by the transmembrane serine protease TMPRSS2. J. Virol..

[124] Koch J., Uckeley Z.M., Doldan P., Stanifer M., Boulant S., Lozach P.Y. (2021). TMPRSS2 expression dictates the entry route used by SARS-CoV-2 to infect host cells. EMBO J..

[125] Bestle D., Heindl M.R., Limburg H., Pilgram O., Moulton H., Stein D.A., Hardes K., Eickmann M., Dolnik O., Rohde C. (2020). TMPRSS2 and furin are both essential for proteolytic activation of SARS-CoV-2 in human airway cells. Life Sci. Alliance.

[126] Matsuyama S., Nao N., Shirato K., Kawase M., Saito S., Takayama I., Nagata N., Sekizuka T., Katoh H., Kato F. (2020). Enhanced isolation of SARS-CoV-2 by TMPRSS2-expressing cells. Proc. Natl. Acad. Sci. USA.

[127] Wilson S.K., Greer B., Hooper J., Zijlstra A., Walker B., Quigley J.P., Hawthorne S. (2005). The membrane-anchored serine protease, TMPRSS2, activates PAR-2 in prostate cancer cells. Biochem. J..

[128] Ko C.-J., Huang C.-C., Lin H.-Y., Juan C.-P., Lan S.-W., Shyu H.-Y., Wu S.-R., Hsiao P.-W., Huang H.-P., Shun C.-T. (2015). Androgen-induced TMPRSS2 activates matriptase and promotes extracellular matrix degradation, prostate cancer cell invasion, tumor growth, and metastasis. Cancer Res..

[129] Lubieniecka J.M., Cheteri M.K., Stanford J.L., Ostrander E.A. (2004). Met160Val polymorphism in the TRMPSS2 gene and risk of prostate cancer in a population-based case-control study. Prostate.

[130] Wang A., Chiou J., Poirion O.B., Buchanan J., Valdez M.J., Verheyden J.M., Hou X., Kudtarkar P., Narendra S., Newsome J.M. (2020). Single-cell multiomic profiling of human lungs reveals cell-type-specific and age-dynamic control of SARS-CoV2 host genes. eLife.

[131] Asselta R., Paraboschi E.M., Mantovani A., Duga S. (2020). ACE2 and TMPRSS2 variants and expression as candidates to sex and country differences in COVID-19 severity in Italy. Aging.

[132] Kehdy F.S., Pita-Oliveira M., Scudeler M.M., Torres-Loureiro S., Zolini C., Moreira R., Michelin L.A., Alvim I., Silva-Carvalho C., Furlan V.C. (2021). Human-SARS-CoV-2 interactome and human genetic diversity: TMPRSS2-rs2070788, associated with severe influenza, and its population genetics caveats in Native Americans. Genet. Mol. Biol..

[133] Singh H., Choudhari R., Nema V., Khan A.A. (2021). ACE2 and TMPRSS2 polymorphisms in various diseases with special reference to its impact on COVID-19 disease. Microb. Pathog..

[134] Pandey R.K., Srivastava A., Singh P.P., Chaubey G. (2022). Genetic association of TMPRSS2 rs2070788 polymorphism with COVID-19 case fatality rate among Indian populations. Infect. Genet. Evol..

[135] Schönfelder K., Breuckmann K., Elsner C., Dittmer U., Fistera D., Herbstreit F., Risse J., Schmidt K., Sutharsan S., Taube C. (2021). Transmembrane serine protease 2 polymorphisms and susceptibility to severe acute respiratory syndrome coronavirus type 2 infection: A German case-control study. Front. Genet..

[136] Monticelli M., Hay Mele B., Benetti E., Fallerini C., Baldassarri M., Furini S., Frullanti E., Mari F., Study G.-C.M., Andreotti G. (2021). Protective role of a TMPRSS2 variant on severe COVID-19 outcome in young males and elderly women. Genes.

[137] Wulandari L., Hamidah B., Pakpahan C., Damayanti N.S., Kurniati N.D., Adiatmaja C.O., Wigianita M.R., Soedarsono, Husada D., Tinduh D. (2021). Initial study on TMPRSS2 p. Val160Met genetic variant in COVID-19 patients. Hum. Genom..

[138] Ravikanth V., Sasikala M., Naveen V., Latha S.S., Parsa K.V.L., Vijayasarathy K., Amanchy R., Avanthi S., Govardhan B., Rakesh K. (2021). A variant in TMPRSS2 is associated with decreased disease severity in COVID-19. Meta Gene.

[139] David A., Parkinson N., Peacock T.P., Pairo-Castineira E., Khanna T., Cobat A., Tenesa A., Sancho-Shimizu V., Casanova J.-L., Abel L. (2022). A common TMPRSS2 variant has a protective effect against severe COVID-19. Curr. Res. Transl. Med..

[140] Torre-Fuentes L., Matías-Guiu J., Hernández-Lorenzo L., Montero-Escribano P., Pytel V., Porta-Etessam J., Gómez-Pinedo U., Matías-Guiu J.A. (2021). ACE2, TMPRSS2, and Furin variants and SARS-CoV-2 infection in Madrid, Spain. J. Med. Virol..

[141] Rokni M., Heidari Nia M., Sarhadi M., Mirinejad S., Sargazi S., Moudi M., Saravani R., Rahdar S., Kargar M. (2022). Association of TMPRSS2 Gene Polymorphisms with COVID-19 Severity and Mortality: A Case-Control Study with Computational Analyses. Appl. Biochem. Biotechnol..

[142] Villapalos-García G., Zubiaur P., Rivas-Durán R., Campos-Norte P., Arévalo-Román C., Fernández-Rico M., Fraile L.G.-F., Fernández-Campos P., Soria-Chacartegui P., de Córdoba-Oñate S.F. (2022). Transmembrane protease serine 2 (TMPRSS2) rs75603675, comorbidity, and sex are the primary predictors of COVID-19 severity. Life Sci. Alliance.

[143] Soker S., Takashima S., Miao H.Q., Neufeld G., Klagsbrun M. (1998). Neuropilin-1 is expressed by endothelial and tumor cells as an isoform-specific receptor for vascular endothelial growth factor. Cell.

[144] Staton C., Kumar I., Reed M., Brown N. (2007). Neuropilins in physiological and pathological angiogenesis. J. Pathol. A J. Pathol. Soc. Great Br. Irel..

[145] He Z., Tessier-Lavigne M. (1997). Neuropilin is a receptor for the axonal chemorepellent Semaphorin III. Cell.

[146] Teesalu T., Sugahara K.N., Kotamraju V.R., Ruoslahti E. (2009). C-end rule peptides mediate neuropilin-1-dependent cell, vascular, and tissue penetration. Proc. Natl. Acad. Sci. USA.

[147] Daly J.L., Simonetti B., Klein K., Chen K.-E., Williamson M.K., Antón-Plágaro C., Shoemark D.K., Simón-Gracia L., Bauer M., Hollandi R. (2020). Neuropilin-1 is a host factor for SARS-CoV-2 infection. Science.

[148] Cantuti-Castelvetri L., Ojha R., Pedro L.D., Djannatian M., Franz J., Kuivanen S., van Der Meer F., Kallio K., Kaya T., Anastasina M. (2020). Neuropilin-1 facilitates SARS-CoV-2 cell entry and infectivity. Science.

[149] Ackermann M., Verleden S.E., Kuehnel M., Haverich A., Welte T., Laenger F., Vanstapel A., Werlein C., Stark H., Tzankov A. (2020). Pulmonary vascular endothelialitis, thrombosis, and angiogenesis in COVID-19. N. Engl. J. Med..

[150] Chapoval S.P., Keegan A.D. (2021). Perspectives and potential approaches for targeting neuropilin 1 in SARS-CoV-2 infection. Mol. Med..

[151] Hashemi S.M.A., Thijssen M., Hosseini S.Y., Tabarraei A., Pourkarim M.R., Sarvari J. (2021). Human gene polymorphisms and their possible impact on the clinical outcome of SARS-CoV-2 infection. Arch. Virol..

[152] Ibrahim W. (2020). Neurological manifestations in coronavirus disease 2019 (COVID-19) patients: A systematic review of literature. CNS Spectr..

[153] Pollock C.E., Sutherland H.G., Maher B.H., Lea R.A., Haupt L.M., Frith A., Macgregor E.A., Griffiths L.R. (2018). The NRP1 migraine risk variant shows evidence of association with menstrual migraine. J. Headache Pain.

[154] Gormley P., Anttila V., Winsvold B.S., Palta P., Esko T., Pers T.H., Farh K.-H., Cuenca-Leon E., Muona M., Furlotte N.A. (2016). Meta-analysis of 375,000 individuals identifies 38 susceptibility loci for migraine. Nat. Genet..

[155] Li Y., Xu J., Chen L., Zhong W.D., Zhang Z., Mi L., Zhang Y., Liao C.G., Bian H.J., Jiang J.L. (2009). HAb18G (CD147), a cancer-associated biomarker and its role in cancer detection. Histopathology.

[156] Pushkarsky T., Zybarth G., Dubrovsky L., Yurchenko V., Tang H., Guo H., Toole B., Sherry B., Bukrinsky M. (2001). CD147 facilitates HIV-1 infection by interacting with virus-associated cyclophilin A. Proc. Natl. Acad. Sci. USA.

[157] Zhang M.-Y., Zhang Y., Wu X.-D., Zhang K., Lin P., Bian H.-J., Qin M.-M., Huang W., Wei D., Zhang Z. (2018). Disrupting CD147-RAP2 interaction abrogates erythrocyte invasion by Plasmodium falciparum. Blood J. Am. Soc. Hematol..

[158] Zhao P., Zhang W., Wang S.J., Yu X.L., Tang J., Huang W., Li Y., Cui H.Y., Guo Y.S., Tavernier J. (2011). HAb18G/CD147 promotes cell motility by regulating annexin II-activated RhoA and Rac1 signaling pathways in hepatocellular carcinoma cells. Hepatology.

[159] Lu M., Wu J., Hao Z., Shang Y., Xu J., Nan G., Li X., Chen Z., Bian H. (2018). Basolateral CD147 induces hepatocyte polarity loss by E-cadherin ubiquitination and degradation in hepatocellular carcinoma progress. Hepatology.

[160] Chen Z., Mi L., Xu J., Yu J., Wang X., Jiang J., Xing J., Shang P., Qian A., Li Y. (2005). Function of HAb18G/CD147 in invasion of host cells by severe acute respiratory syndrome coronavirus. J. Infect. Dis..

[161] Shilts J., Crozier T.W., Greenwood E.J., Lehner P.J., Wright G.J. (2021). No evidence for basigin/CD147 as a direct SARS-CoV-2 spike binding receptor. Sci. Rep..

[162] Fenizia C., Galbiati S., Vanetti C., Vago R., Clerici M., Tacchetti C., Daniele T. (2021). SARS-CoV-2 Entry: At the Crossroads of CD147 and ACE2. Cells.

[163] Geng J., Chen L., Yuan Y., Wang K., Wang Y., Qin C., Wu G., Chen R., Zhang Z., Wei D. (2021). CD147 antibody specifically and effectively inhibits infection and cytokine storm of SARS-CoV-2 and its variants delta, alpha, beta, and gamma. Signal Transduct. Target. Ther..

[164] Mao Y., Yan J., Wang C., Wang Z., Liu P., Yuan W. (2014). CD147 expression level and rs8259 T/A polymorphism of CD147 in patients with acute coronary syndrome. Zhonghua Xin Xue Guan Bing Za Zhi.

[165] Li M.-P., Hu X.-L., Yang Y.-L., Zhang Y.-J., Zhou J.-P., Peng L.-M., Tang J., Chen X.-P. (2017). Basigin rs8259 polymorphism confers decreased risk of chronic heart failure in a Chinese population. Int. J. Environ. Res. Public Health.

[166] Latini A., Agolini E., Novelli A., Borgiani P., Giannini R., Gravina P., Smarrazzo A., Dauri M., Andreoni M., Rogliani P. (2020). COVID-19 and genetic variants of protein involved in the SARS-CoV-2 entry into the host cells. Genes.

[167] Crosnier C., Bustamante L.Y., Bartholdson S.J., Bei A.K., Theron M., Uchikawa M., Mboup S., Ndir O., Kwiatkowski D.P., Duraisingh M.T. (2011). Basigin is a receptor essential for erythrocyte invasion by Plasmodium falciparum. Nature.

[168] Xiong L., Edwards C.K., Zhou L. (2014). The biological function and clinical utilization of CD147 in human diseases: A review of the current scientific literature. Int. J. Mol. Sci..

[169] Gudowska-Sawczuk M., Mroczko B. (2021). The Role of Neuropilin-1 (NRP-1) in SARS-CoV-2 Infection: Review. J. Clin. Med..

[170] Petersen D., Steyl C., Scholtz D., Baker B., Abdullah I., Uren C., Möller M. (2022). African Genetic Representation in the Context of SARS-CoV-2 Infection and COVID-19 Severity. Front. Genet..

[171] Mostafa-Hedeab G. (2020). ACE2 as drug target of COVID-19 virus treatment, simplified updated review. Rep. Biochem. Mol. Biol..

[172] Vitiello A., Ferrara F., Porta R. (2021). Remdesivir and COVID-19 infection, therapeutic benefits or unnecessary risks?. Ir. J. Med. Sci..

[173] Charoute H., Elkarhat Z., Elkhattabi L., El Fahime E., Oukkache N., Rouba H., Barakat A. (2022). Computational screening of potential drugs against COVID-19 disease: The Neuropilin-1 receptor as molecular target. VirusDisease.

[174] Yamamoto M., Kiso M., Sakai-Tagawa Y., Iwatsuki-Horimoto K., Imai M., Takeda M., Kinoshita N., Ohmagari N., Gohda J., Semba K. (2020). The anticoagulant nafamostat potently inhibits SARS-CoV-2 S protein-mediated fusion in a cell fusion assay system and viral infection in vitro in a cell-type-dependent manner. Viruses.

[175] Uddin H., Zonder J.A., Azmi A.S. (2020). Exportin 1 inhibition as antiviral therapy. Drug Discov. Today.

[176] Yagin F.H., Cicek İ.B., Alkhateeb A., Yagin B., Colak C., Azzeh M., Akbulut S. (2023). Explainable artificial intelligence model for identifying COVID-19 gene biomarkers. Comput. Biol. Med..

